# Whole Genome Sequencing of *Mycobacterium africanum* Strains from Mali Provides Insights into the Mechanisms of Geographic Restriction

**DOI:** 10.1371/journal.pntd.0004332

**Published:** 2016-01-11

**Authors:** Kathryn Winglee, Abigail Manson McGuire, Mamoudou Maiga, Thomas Abeel, Terrance Shea, Christopher A. Desjardins, Bassirou Diarra, Bocar Baya, Moumine Sanogo, Souleymane Diallo, Ashlee M. Earl, William R. Bishai

**Affiliations:** 1 Center for Tuberculosis Research, Department of Medicine, Johns Hopkins University, Baltimore, Maryland, United States of America; 2 Genome Sequencing and Analysis Program, The Broad Institute of MIT & Harvard, Cambridge, Massachusetts, United States of America; 3 Project SEREFO (Centre de Recherche et de Formation sur le VIH/Sida et la Tuberculose)/University of Sciences, Technics and Technologies of Bamako (USTTB), Bamako, Mali; 4 Delft Bioinformatics Lab, Delft University of Technology, Delft, The Netherlands; University of Tennessee, UNITED STATES

## Abstract

**Background:**

*Mycobacterium africanum*, made up of lineages 5 and 6 within the *Mycobacterium tuberculosis* complex (MTC), causes up to half of all tuberculosis cases in West Africa, but is rarely found outside of this region. The reasons for this geographical restriction remain unknown. Possible reasons include a geographically restricted animal reservoir, a unique preference for hosts of West African ethnicity, and an inability to compete with other lineages outside of West Africa. These latter two hypotheses could be caused by loss of fitness or altered interactions with the host immune system.

**Methodology/Principal Findings:**

We sequenced 92 MTC clinical isolates from Mali, including two lineage 5 and 24 lineage 6 strains. Our genome sequencing assembly, alignment, phylogeny and average nucleotide identity analyses enabled us to identify features that typify lineages 5 and 6 and made clear that these lineages do not constitute a distinct species within the MTC. We found that in Mali, lineage 6 and lineage 4 strains have similar levels of diversity and evolve drug resistance through similar mechanisms. In the process, we identified a putative novel streptomycin resistance mutation. In addition, we found evidence of person-to-person transmission of lineage 6 isolates and showed that lineage 6 is not enriched for mutations in virulence-associated genes.

**Conclusions:**

This is the largest collection of lineage 5 and 6 whole genome sequences to date, and our assembly and alignment data provide valuable insights into what distinguishes these lineages from other MTC lineages. Lineages 5 and 6 do not appear to be geographically restricted due to an inability to transmit between West African hosts or to an elevated number of mutations in virulence-associated genes. However, lineage-specific mutations, such as mutations in cell wall structure, secretion systems and cofactor biosynthesis, provide alternative mechanisms that may lead to host specificity.

## Author Summary

*Mycobacterium africanum* consists of two lineages, lineages 5 and 6, within the *Mycobacterium tuberculosis* complex (MTC) that cause human tuberculosis in West Africa, but are found rarely outside of this region. Our analysis of the whole genome sequences of 26 lineage 5 and 6 isolates, and 66 isolates from other lineages within the MTC, reveal that *M*. *africanum* does not meet modern criteria to be considered an independent species. We analyzed drug resistance-associated genes and found that drug resistance evolves within these lineages through similar mechanisms as observed for the rest of the MTC in Mali. Though we did not see an elevated number of mutations in virulence-associated genes in these two lineages, we identified a number of lineage-specific mutations, pseudogenes and changes in gene content that may impact virulence and host specificity, and improve, overall, our understanding of what make these lineages unique.

## Introduction

*Mycobacterium africanum* is a member of the *Mycobacterium tuberculosis* complex (MTC) that causes up to half of all tuberculosis cases in West Africa [1]. First identified by Castets in 1968, it was originally characterized as having biochemical characteristics intermediate between *Mycobacterium tuberculosis*, which consists of lineages 1, 2, 3, 4, and 7 and is the main cause of human tuberculosis, and *Mycobacterium bovis*, an animal-adapted lineage that causes bovine tuberculosis [2]. Later work divided *M*. *africanum* into two lineages, *M*. *africanum* West African type I and *M*. *africanum* West African type II, which became known as lineages 5 and 6, respectively, within the MTC [3, 4].

Lineages 5 and 6 cause a disease similar to classically defined *M*. *tuberculosis*, although it has been suggested that human disease caused by these lineages may differ compared to that caused by lineages 1–4. For example, patients with lineage 6 disease have been reported to show attenuated ESAT-6 responses compared to patients with classical *M*. *tuberculosis* lineage disease [5, 6]. In addition, in liquid culture systems it has been reported that *M*. *africanum* has a slower growth rate with a larger bacillary size than *M*. *tuberculosis* [7, 8]. While some studies have found that *M*. *africanum* is less virulent than *M*. *tuberculosis*, both in animal models and human patients [7, 9–11], others show that there is no difference [12]. Though these contradicting results may be due to differences in the study populations, they underscore how little is known about lineages 5 and 6.

Contributing to this lack of knowledge, while lineages 1–4 are widely distributed around the globe, lineages 5–7 are limited to certain regions of Africa [13]. Lineage 7 has only been found in Ethiopia [14], and lineages 5 and 6 are found almost exclusively in patients living in West Africa, with very few cases occurring outside of this region, mostly involving recent immigrants from West Africa [1]. The reason for the apparent geographic restriction of lineages 5 and 6 is unknown. One hypothesis is the presence of an undiscovered animal reservoir endemic to West Africa, which is supported by the close relationship between lineages 5 and 6 and the animal-adapted lineages of the MTC [15, 16]. Another hypothesis is that lineages 5 and 6 have a unique predilection for humans with genetic backgrounds common in West Africa. In fact, using a retrospective epidemiological study of the MTC in Ghana, Asante-Poku et al. showed that lineage 5 is associated with the Ewe ethnic group [17]. A third hypothesis is that lineages 5 and 6 are unable to compete with other lineages outside of West Africa, either due to loss of fitness or decreased transmissibility, thus explaining their limited global distribution [7].

Historically, mycobacterial subspecies were defined by biochemical assays, but, as genetic tools became more readily available, it is now possible to identify genomic regions that define MTC lineages [18]. The publication of the whole genome sequence of *M*. *africanum* GM041182, a single lineage 6 strain, provided valuable insights into the genetics of this lineage [19]. For instance, the authors identified lineage 6-specific pseudogenes, a novel region not present in *M*. *tuberculosis*, and single nucleotide polymorphisms (SNPs) in key genes, all of which may play a role in the geographic restriction of lineage 6. A later study sequenced four additional lineage 6 isolates and was able to confirm many of these findings, but also showed that not all mutations identified in *M*. *africanum* GM041182 are shared by other members of this lineage [8]. To our knowledge, no study has closely analyzed the genetics of lineage 5.

From these studies, it is clear that more sequenced isolates are needed to fully characterize the genetics of lineages 5 and 6 and to illuminate mechanisms that may explain its geographic isolation. Toward this end, we sequenced 92 clinical MTC isolates from Mali, a country in West Africa in which 26.2% and 1.6% of tuberculosis cases are caused by lineage 6 and lineage 5, respectively [20] [1]. Using these and previously published data, we performed both alignment- and assembly-based comparative analyses to further refine our understanding of lineage-specific genomic features that might explain the geographic distribution of lineages 5 and 6. To our knowledge, this is the largest collection of lineage 6 strains sequenced to date, and the first in depth whole genomic characterization of lineage 5.

## Materials and Methods

### Samples

101 strains were selected from clinical isolates collected in Bamako, Mali [20], and included all strains identified by spoligotyping as *M*. *africanum*, *M*. *tuberculosis* T1, or *M*. *bovis*. Of these strains, 92 were still viable and were submitted for whole genome sequencing. These 92 strains will be referred to as the “Mali Collection” (S1A Table). In addition, to improve MTC lineage representation, we selected additional whole genome assemblies that matched the quality of our assemblies. These included four finished *M*. *bovis* genomes available from GenBank (*M*. *bovis* AF2122/97 [21], *M*. *bovis* BCG Mexico [22], *M*. *bovis* BCG Pasteur 1173P2 [23], and *M*. *bovis* BCG Tokyo 172 [24]), a set of 40 *M*. *tuberculosis* strains (9 lineage 1 strains, 12 lineage 2 strains, 7 lineage 3 strains, and 12 lineage 4 strains) from South Africa [25], the finished *M*. *africanum* genome from Genbank (*M*. *africanum* GM041182) [19], and our outgroup, *M*. *canettii* CIPT 140010059 [26]. Combined with the Mali Collection, these 137 strains will be referred to as the “Assembly Collection” (S1B Table). Finally, all 161 strains (122 lineage 2, two lineage 3, and 37 lineage 4) from a study in China were included in the variant analysis to improve geographical and lineage representation [27]. The samples from the China study (S1C Table) combined with the samples from South Africa and Mali (for a total of 289 strains) will be referred to as the “Alignment Collection”.

### Ethics statement

The study protocols for the Mali samples were approved by the Ethics Committee of the University of Bamako and the Institutional Review Board of the National Institute of Allergy and Infectious Diseases, National Institutes of Health (NIAID/NIH), Bethesda, MD, USA. For all samples, written informed consent was obtained from study participants prior to cohort enrollment [20]. For the South African samples, Biomedical Research Ethics Council (BREC) approval from the University of KwaZulu-Natal was granted for collection of sputum specimens from study participants and for whole genome sequencing of clinical strains. Written informed consent was obtained from study participants prior to cohort enrollment, or waived by BREC [25].

### Drug susceptibility testing

Drug resistance to isoniazid, rifampicin, ethambutol and streptomycin was tested for all Mali strains as previously described [20]. We confirmed those results by submitting 17 strains to National Jewish Health in Colorado for agar proportion testing of isoniazid, rifampicin, ethambutol, ofloxacin, niacin, kanamycin, ethionamide, capreomycin, amikacin, cycloserine and para-aminosalicylic acid, as well as radiometric testing of ciprofloxacin and pyrazinamide. The agar proportion results confirmed the mycobacterial growth indicator tube (MGIT) tests performed in Mali. Genotypic drug resistance was determined for rifampicin, isoniazid, ethambutol, streptomycin, ofloxacin, kanamycin and ethionamide using genetic markers from line-probe assays (S2 Table).

### Genome sequencing

Extraction of genomic DNA was performed on 10 mL cultures grown in 7H9 broth using the CTAB-lysozyme method as previously described [28]. Library preparation and whole genome sequencing (WGS) were performed as previously described [29–31]. GenBank accessions for all strains used in this analysis can be found in S1B Table, along with assembly statistics for the new sequences generated at the Broad Institute (92 sequences from Mali generated for this study, and 40 sequences from South Africa).

### Annotation

All genomes in our Assembly Collection were uniformly annotated by transferring annotations from *M*. *tuberculosis* H37Rv. The reference *M*. *tuberculosis* H37Rv genome (accession CP003248.2) was aligned to draft assemblies using Nucmer [32]. This alignment was used to map reference genes over to the target genomes. Using this methodology, annotations were successfully transferred onto all 137 strains for 3466 of the *M*. *tuberculosis* H37Rv genes; the rest of the *M*. *tuberculosis* H37Rv genes transferred to a subset of the genomes.

For those genes not cleanly mapping to *M*. *tuberculosis* H37Rv, the protein-coding genes were predicted with the software tool Prodigal [33]. tRNAs were identified by tRNAscan-SE [34] and rRNA genes were predicted using RNAmmer [35]. Gene product names were assigned based on top blast hits against the SwissProt protein database (> = 70% identity and > = 70% query coverage), and protein family profile search against the TIGRfam hmmer equivalogs. Additional annotation analyses performed include Pfam [36], TIGRfam [37], Kyoto Encyclopedia of Genes and Genomes (KEGG) [38], clusters of orthologous groups (COG) [39], Gene Ontology (GO) [40], enzyme commission (EC) [41], SignalP [42], and Transmembrane Helices; Hidden Markov Model (TMHMM) [43].

### Digital spoligotyping

Reads from each isolate were aligned against the 43 spacer sequences traditionally used in wet lab spoligtyping [28, 44]. From these alignments, the number of matching reads was used to determine if the spacer was present. The spacer was considered absent if the read count total was in the lowest quartile of counts. Spacers were defined as present by using a Bonferroni corrected p-value based on an exponential distribution of the average absent spacer counts. If the p-value was <0.01 the spacer was considered to be present. The spacer pattern was matched to the SITVITWEB database to generate a named spoligytpe for each isolate and to determine the spoligotype international type (SIT) [45].

### Orthogroup clustering and phylogenetic trees

SYNERGY2 [46–48], available at http://sourceforge.net/projects/synergytwo/, was used to identify cluster-based orthogroups across our Assembly Collection of 137 genomes, which we will refer to as “SYNERGY orthogroups”. In addition, for each *M*. *tuberculosis* H37Rv gene, we defined a second set of annotation transfer-based ortholog groups as the set of genes for which annotations were transferred from this *M*. *tuberculosis* H37Rv gene in our annotation protocol, which we will refer to as “*M*. *tuberculosis* H37Rv-based orthologs”. Genes without *M*. *tuberculosis* H37Rv orthologs were manually examined in the context of their SYNERGY orthogroups to identify lineage-specific novel genes.

Phylogenetic trees were generated by applying RAxML [49] to a concatenated alignment of 3343 single-copy core SYNERGY orthogroups (excluding orthogroups with paralogs) across all 137 organisms. Bootstrapping was performed using RAxML’s rapid bootstrapping algorithm (1000 iterations).

### Average nucleotide identity analysis (ANI)

Calculations of ANI were done as previously described [50, 51] using the SYNERGY orthogroups calculated from the Assembly Collection.

### Gene content analysis

PAUP [52] was used to reconstruct gain and loss of *M*. *tuberculosis* H37Rv-based orthologs at ancestral nodes of the Assembly Collection phylogenetic tree using parsimony. In order to analyze changes in gene content, we used a cost matrix with values of 10 for a gene gain, 5 for a gene loss, and 0.2 for an increase or decrease in copy number. We looked for orthologs found within all members of one clade, and absent in other clades. As a further filter, we also required that orthogroups be found in >80% of the clade of interest, and <20% of other strains. We performed this analysis for four key clades: lineage 5, lineage 6, the clade including *M*. *bovis* and lineage 6, and the clade including lineages 5, 6 and *M*. *bovis*.

In addition, we selected the Pfam gene categories most expanded or reduced in each clade of interest. We determined significance using Fisher’s test (Q<0.05). For each of the clades described above, we compared the strains below this node versus all other strains in our analysis.

### Identification of SNPs

For our Alignment Collection, reads were mapped onto a reference strain of *M*. *tuberculosis* H37Rv (GenBank accession number CP003248.2) using BWA version 0.5.9.9 [53]. In cases where read coverage of the reference was greater than 200x, reads were down-sampled using Picard [54] prior to mapping. Variants, including both single nucleotide polymorphisms (SNPs) and multi-nucleotide polymorphisms, were identified using Pilon version 1.5 as described [29] and were used to construct phylogenetic trees using FastTree [55].

We defined lineage-specific variants for lineage X, as those occurring in at least 95% of the strains of lineage X (true positive rate >95%), missing in less than 5% of strains of lineage X (positive predictive value >95%), occurring in less than 5% of the strains that do not belong to lineage X (true negative rate >95%) and not occurring in at least 95% of strains not belonging to lineage X (negative predictive value >95%). The absolute number of true positives must exceed seven. Formulas are schematically presented in S3 Table.

Mutations were considered *M*. *africanum*-specific (lineage 5 and 6-specific, identified as LIN-Maf in S4 and S5 Tables) if they met these cutoffs for lineage 5 and 6 combined, and were present in both lineage 5 strains. Similarly, mutations were considered *M*. *tuberculosis-*specific if they met these cutoffs for lineages 1–4 combined. No *M*. *tuberculosis-*specific mutations were identified. Due to inclusion of only two lineage 5 strains in our dataset, no lineage-specific variants were identified in lineage 5. Thus, for this lineage only, we used a less stringent requirement to define lineage-specific variants: we required that variants be present in both lineage 5 strains and in <5% of the strains in each other lineage. We classified each gene containing a lineage-specific variant into functional group categories, including GO [40], KEGG [38], Pfam [36], and COG [39]. We then evaluated enrichment using Fisher's Exact test and corrected for multiple comparisons using the Storey method for functional group categories [56].

### Identification of pseudogenes

A pseudogene was defined as any gene that had a loss of function mutation anywhere within the coding sequence. Loss of function mutations were defined as nonsense mutations, or insertions or deletions with lengths that were not multiples of 3 base pairs or were greater than 30 base pairs. Lineage-specific pseudogenes were determined using the same definitions as for variants on a per gene basis (positive predictive value > 95%, negative predictive value >95%, true positive rate >95%, true negative rate >95%, number of true positives >7, with the exception of lineage 5, which used the SNP cutoffs of pseudogene in both lineage 5 strains and in <5% in each other lineage).

### Computational gene function assessments

The effect of select non-synonymous mutations on protein function was assessed using the online version of SIFT at default settings [57], unless there was low confidence in the prediction, in which case SIFT was run for each of the four available databases (UniRef90 from April 2011 [default], UniProt-SwissProt 57.15 from April 2011, UniProt-TrEMBL from March 2009 and NCBI nonredundant from March 2011). Peptide binding was predicted using the NetMHCII online tool with default settings [58].

## Results

### *M*. *africanum* and *M*. *tuberculosis* lineages are part of the same species

**Fig 1:**
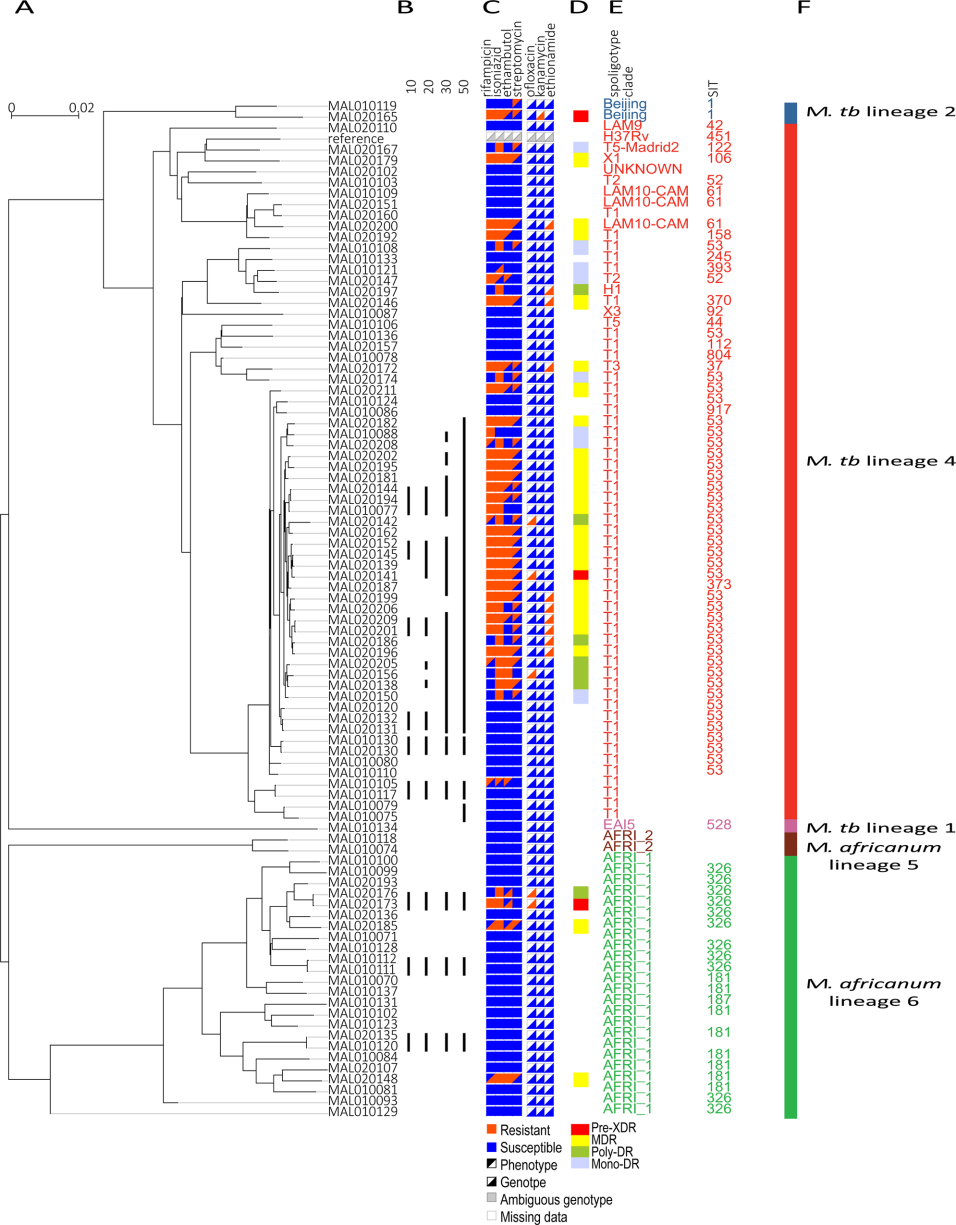
*M*. *africanum* and *M*. *tuberculosis* drug resistance is genetically similar. A) SNP-based phylogenetic tree of 92 newly sequenced strains from Mali (the Mali Collection), constructed using FastTree [55]. B) Groups differing by 10, 20, 30, or 50 SNPs are connected with black bars, as calculated in Cohen et al. [25]. C) Comparison of genotypic and phenotypic DST (drug susceptibility testing). Genotypic drug resistance was calculated using a list of mutations known to confer drug resistance (S2A Table) [59, 60]. D) Drug resistance category (mono-DR, poly-DR, MDR, or pre-XDR) based on genotype. E) Digital spoligotype clade and F) digital spoligotype international type (SIT), colored by lineage. F) The SIT is blank if the SITVITWEB database [45] did not contain a SIT for that strain’s digital spoligotype pattern.

**Fig 2:**
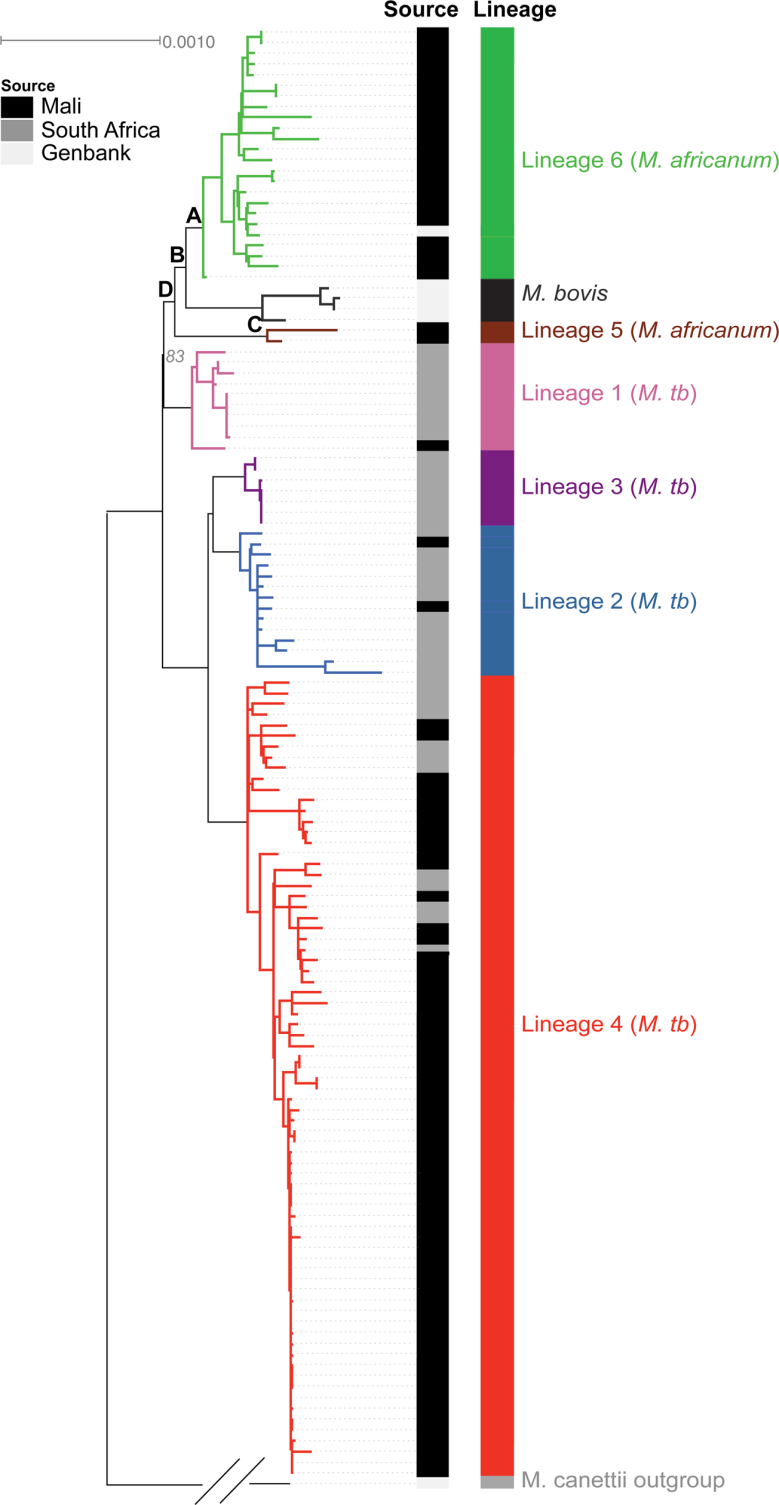
Phylogenetic tree of 92 newly sequenced strains from Mali, together with 45 additional strains with whole-genome assemblies (the Assembly Collection). Nodes, lineages, and newly-sequenced Mali strains are indicated. All key nodes separating the major lineages had bootstrap values of 100%, except for the node separating *M*. *tuberculosis* lineage 1 and *M*. *africanum* lineage 5, which had a bootstrap value of 83%. Letters indicate key nodes analyzed in detail: (A) lineage 6, (B) the clade including *M*. *bovis* and lineage 6, (C) lineage 5, and (D) the clade including lineages 5, 6 and *M*. *bovis*.

Our collection of 92 clinical MTC strains was isolated from patients presenting with pulmonary tuberculosis at Point G, Bamako, Mali between 2006 and 2010 as part of a cross-sectional study to analyze the diversity of the MTC in Mali [20]. All patients were Mali natives, with the exception of one patient born in central Africa (S1A Table). We sequenced this collection using the Illumina sequencing platform, and the resulting reads were both assembled into contigs and aligned against the *M*. *tuberculosis* H37Rv reference genome. Based on our phylogenetic reconstructions, our collection included one lineage 1, two lineage 2, zero lineage 3, 63 lineage 4, two lineage 5 and twenty-four lineage 6 strains (Fig 1). The spoligotype distribution of our collection is representative of what has previously been observed in West Africa, except that we had a higher proportion of SIT53 (T1) strains and a lower proportion of SIT181 (AFRI_1) strains (Figs 1 and S1) [45]. In order to perform statistical comparisons of the *M*. *tuberculosis*, *M*. *africanum* and *M*. *bovis* lineages, our newly sequenced dataset (the “Mali Collection”) was combined with data from additional strains from GenBank and South Africa (“Assembly Collection”, Fig 2), as well as data from China (“Alignment Collection”; see Materials and Methods and S1 Table). These additional comparator genomes enabled us to examine in detail the distinguishing characteristics of lineages 5 and 6 that might explain their geographic restriction.

Since this represents the largest collection of whole genome sequences of lineage 5 and 6 strains to date, we used our Assembly Collection to conduct a detailed examination of their phylogeny and characteristics in relation to other members of the MTC, including *M*. *bovis* and *M*. *tuberculosis*. *M*. *bovis* is considered an animal strain that mainly infects cattle and rarely humans, while *M*. *tuberculosis* is human adapted, and lineages 5 and 6 are thought to be intermediate between the two [1, 15]. Using our Assembly Collection, we constructed a high-resolution phylogenetic tree using 3,343 single-copy core orthogroups (sets of orthologs) conserved across all 137 strains (Materials and Methods). This tree was rooted using the outgroup *M*. *canettii* and agreed with phylogenies observed in other studies, including the fact that each of the lineages was clearly separated from the other, with lineage 5 being more closely related to human-adapted strains and lineage 6 being more closely related to *M*. *bovis* (Fig 2) [13, 15].

**Fig 3:**
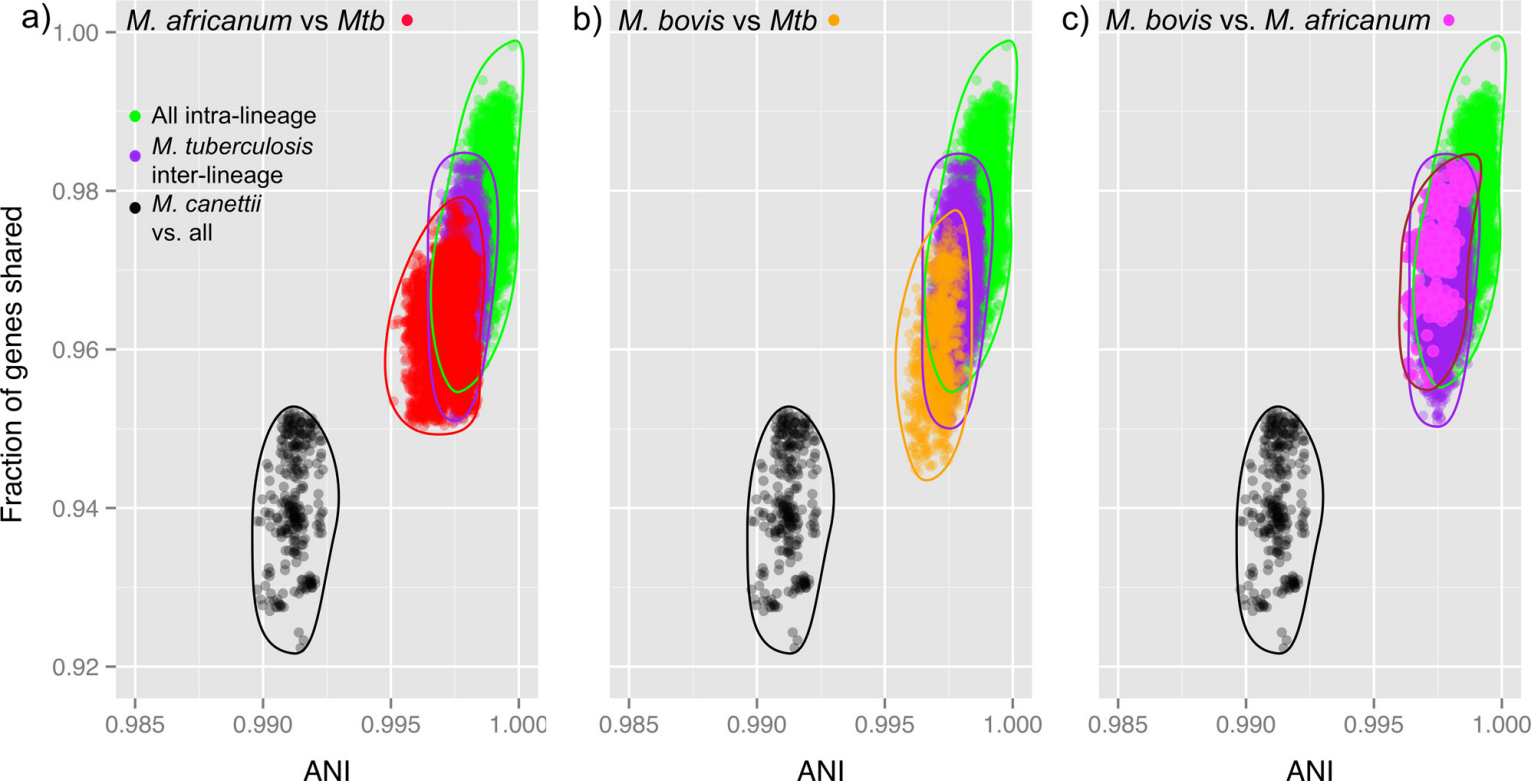
Average nucleotide identity (ANI) analysis indicates *M*. *africanum* and *M*. *tuberculosis* are not separate species. A) ANI values when comparing *M*. *africanum* and *M*. *tuberculosis* do not cross the ANI species threshold of 94–95%. In fact, this comparison shows that the distribution of *M*. *africanum*/*M*. *tuberculosis* comparisons (red) overlaps that of inter-lineage *M*. *tuberculosis* comparisons (purple), indicating that *M*. *africanum* should be considered another lineage of *M*. *tuberculosis*. B) Similarly, ANI values when comparing *M*. *bovis* and *M*. *tuberculosis* also overlap with inter-lineage *M*. *tuberculosis*, and indicate that *M*. *bovis* should also be considered another lineage of *M*. *tuberculosis*. C) ANI values comparing *M*. *africanum* and *M*. *bovis* (pink) also overlap inter-lineage *M*. *tuberculosis* comparisons (green).

It has been previously shown, using average nucleotide identity (ANI) analysis, that separate bacterial species share <65–90% of genes and have no more than 94–95% ANI among shared genes [50, 51]. Using gene content and nucleotide variation among shared genes, we examined the genetic distances between strains within the Assembly Collection to understand how mycobacterial species fit within this framework. In agreement with previous studies showing the close relationship between MTC subspecies, including *M*. *africanum*, we observed that there was little diversity between the lineages analyzed [61]. Strikingly, values from inter-lineage comparisons of *M*. *tuberculosis*, *M*. *bovis*, and *M*. *africanum* strains overlapped those from intra-lineage comparisons, showing very little separation, with >99% ANI and >94% fraction of shared genes (Fig 3). These results are in agreement with previous observations that these different organisms should not, in fact, be named different species [61].

In contrast, MTC pairwise comparisons with *M*. *canettii* revealed a clear separation between the two groups, suggesting that they occupy distinct niches (Fig 3). *M*. *canettii* is a smooth tubercle bacilli that causes human tuberculosis in East Africa and is considered an emerging pathogen in some parts of the world, but its natural host(s) and reservoirs remain unknown [62]. Thus, it might be argued, based on these data and the traditional cutoffs set by ANI analysis, that all MTC members should be named the same species, and that even *M*. *canettii* should be included since pairwise identities with MTC exceeded these thresholds (Fig 3). However, as Smith et al. have previously discussed [61] changes in nomenclature can cause confusion in the literature, and so we will continue to refer to *M*. *africanum*-associated lineages as either lineage 5 or 6 within the MTC.

### Lineage 6 is as diverse as lineage 4 strains and involved in recent person-to-person transmission

**Fig 4:**
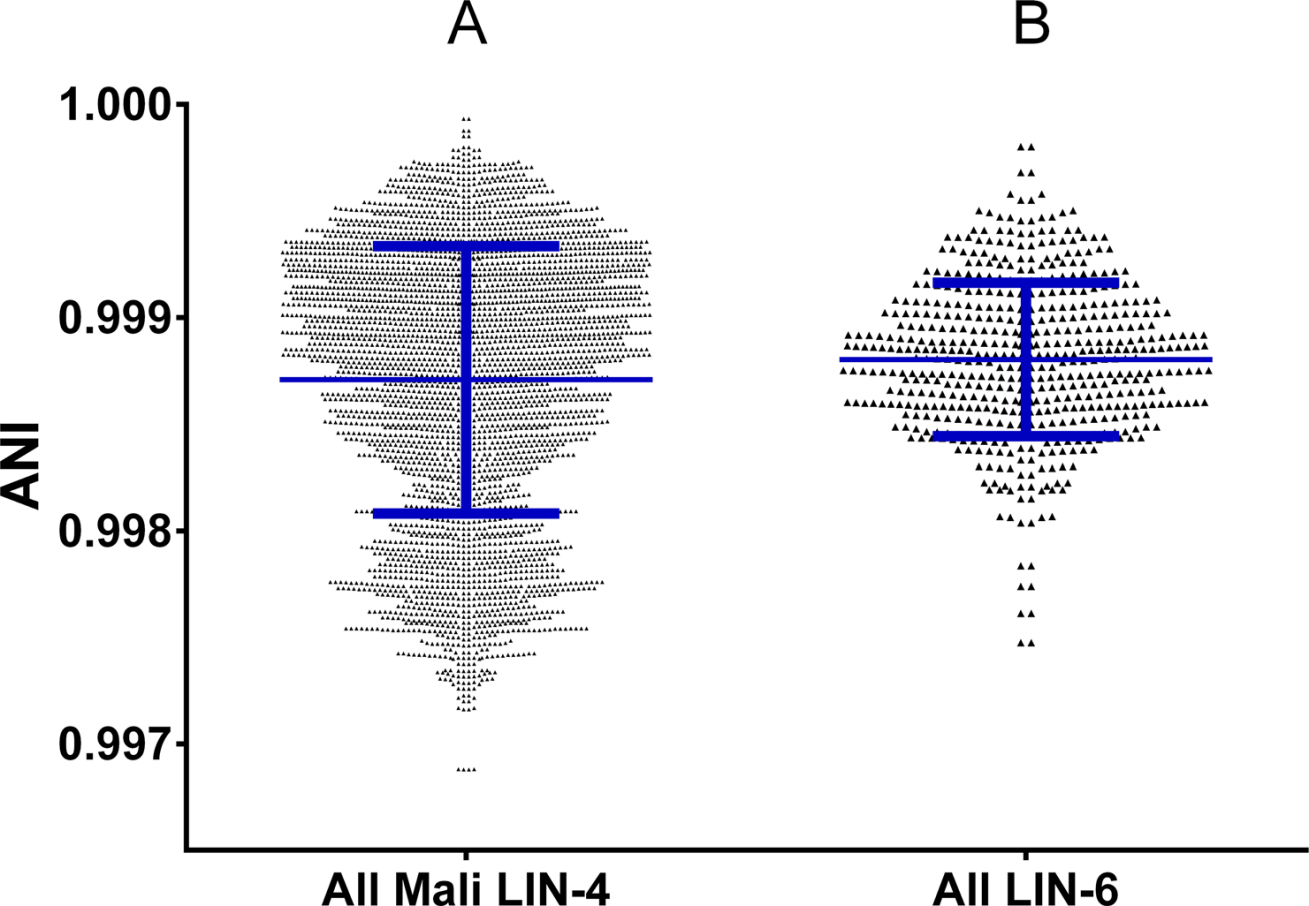
Diversity in Mali lineage 4 and lineage 6 strains. ANI values for comparisons (A) within all Mali lineage 4 isolates and (B) within all lineage 6 isolates. Blue lines indicate mean ± standard deviation. The means of these two groups was not significantly different using the Mann-Whitney test.

Despite the fact that lineages 5 and 6 are so closely related to lineages 1–4, as demonstrated by Fig 3, they are still unique in being geographically restricted compared to these other lineages. One hypothesis for this restriction is that they are less fit, unable to compete with other lineages within the MTC. To examine this possibility, we looked within the Mali Collection for clues that lineage 6 strains were not as diverse as strains from lineage 4, the other predominant lineage within the region. We analyzed the breadth of pairwise diversity within lineage 6 using the ANI output and compared this diversity to that of lineage 4 strains isolated within Mali. ANI diversity was not statistically different when comparing these two groups of strains (Fig 4). Although this result does not eliminate the possibility of differing ecologies, such as an animal reservoir for lineage 6, as has previously been hypothesized [16], it does suggest that lineage 6 has not undergone a recent selective sweep or population bottleneck that would make lineage 6 populations circulating within Mali less diverse than lineage 4 populations [63].

In addition to being diverse, we also observed highly similar lineage 6 strains among this collection. Three pairs of lineage 6 strains were separated by less than 10 SNPs relative to *M*. *tuberculosis* H37Rv (Fig 1B; see Materials and Methods), including isolates from both HIV-positive and immunocompetent patients. There were also six such clusters within lineage 4. A cutoff of 12 SNPs has previously been used to determine recent transmission [64]. Thus, strains separated by less than 10 SNPs provide evidence of transmission, suggesting that 6 of 24 (25%) of our lineage 6 strains and 13 of 63 (21%) of our lineage 4 strains were involved in recent transmission events, confirming previous observations based on alternative genotyping approaches that there is robust ongoing transmission of lineage 6 within this region [9].

### Lineages 5 and 6 are not enriched for mutations in genes associated with virulence

**Fig 5:**
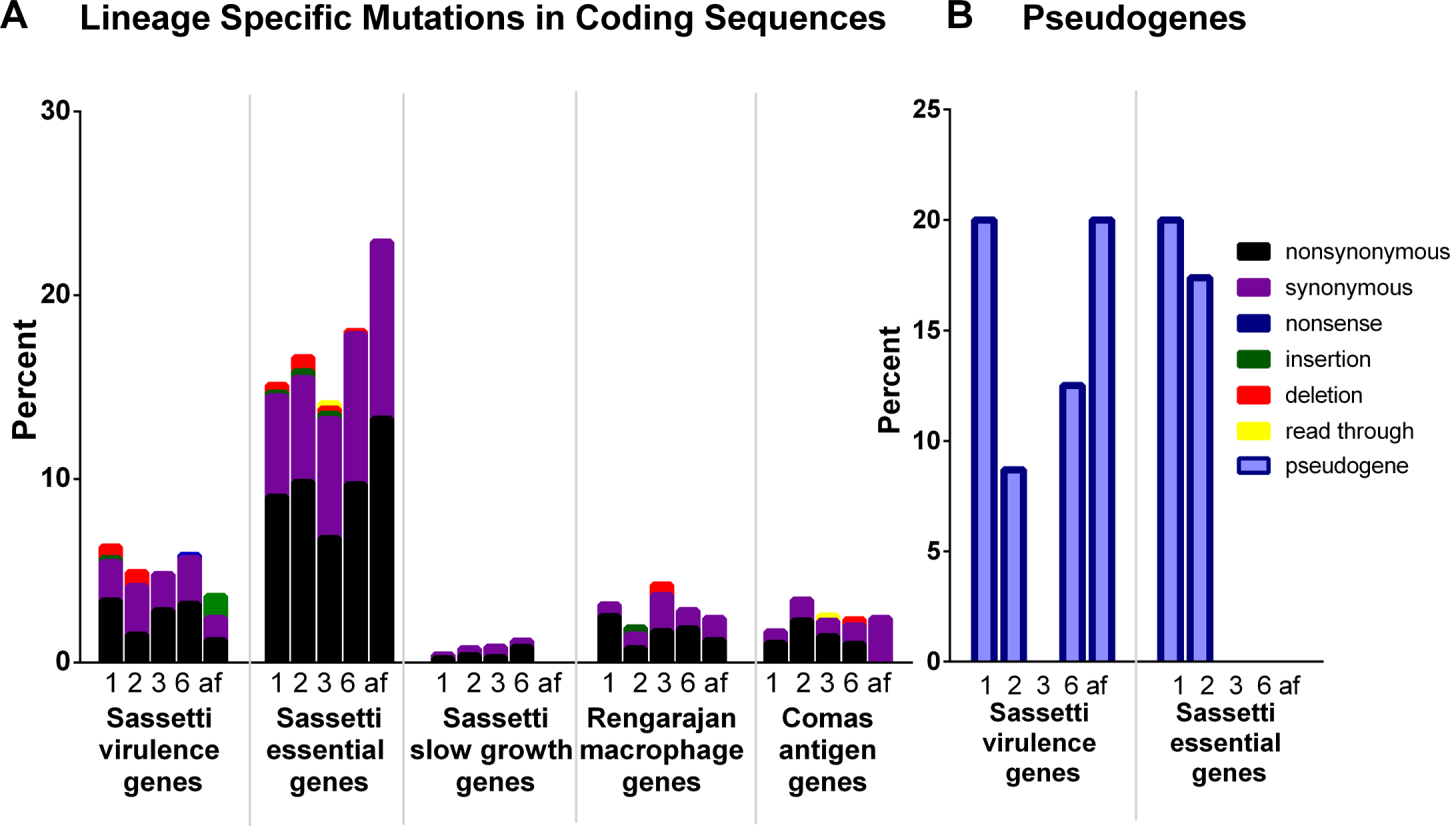
Percentage of lineage-specific mutations in virulence associated genes. A) Percentage of lineage-specific mutations in coding sequences of the genes in each category. Sassetti virulence genes are genes that were identified in [66] as being required for virulence in mice. Sassetti essential and slow growth genes were identified by Sassetti et al. under *in vitro* conditions using TraSH [65]. Rengarajan macrophage genes were identified by Rengarajan et al. as being required for growth in macrophages [67]. Comas antigen genes were genes identified by Comas et al. as containing T cell epitopes [4]. The color of the bar indicates type of mutation. B) Percentage of lineage-specific pseudogenes falling into the above defined categories. Missing categories had no pseudogenes in any lineage. Lineage is indicated by the number below each bar, while ‘af’ indicates mutations found in both lineages 5 and 6 (both *M*. *africanum* lineages).

Given the reports of lineages 5 and 6 strains having decreased virulence [7, 9–11], we hypothesized that altered virulence may contribute to geographical restriction, either due to changes in host requirements or to a reduction in fitness. To test this hypothesis, we examined lineage-specific pseudogenes (truncated genes) and non-synonymous SNPs in known essential genes, slow growth genes, and genes required for virulence in mice and growth in macrophages to determine whether lineages 5 and 6 had an enrichment of defects in these genes that might contribute to overall altered virulence [65–67]. Although both lineages 5 and 6 had lineage-specific mutations in these gene categories, so did other lineages (S4A and S5 Tables), and the proportion of mutated genes in lineage 6 was not significantly different from that of the other MTC lineages [8] (Fig 5). Lineage 4 was not included on this graph because it only had one lineage-specific mutation in an intergenic region when aligned to *M*. *tuberculosis* H37Rv, which is a member of lineage 4, and lineage 5 was excluded due to low sample size. We performed a similar analysis on the full length of genes encoding known T cell antigens as defined by Comas et al. [4] to explore whether alterations in these genes might be restricting host specificity, but again we observed no significant difference in the proportion of lineage 6-specific mutations that fell within these genes as compared to lineages 1, 2 and 3 (Fig 5). Similarly, we looked for enrichment of lineage-specific mutations in COG, GO, KEGG, Pfam and TIGRfam gene categories, but found no enrichment in any of these categories, either for pseudogenes or non-synonymous SNPs (Q > 0.05). These results corroborate our observations from ANI that the lineages of the MTC are very similar in their overall genetic composition and suggest that lineage 6 is not enriched for mutations in virulence genes relative to other lineages. However, while the overall number of mutations in virulence genes was not enriched, we identified mutations in these genes that might have an impact on virulence that will be discussed below.

### Lineage 6 evolves drug resistance through similar mechanisms to other MTC lineages

Studies have shown that lineages 5 and 6 evolve drug resistance less often compared to other MTC lineages, including the study from which these sequenced strains were obtained [20, 68]. Thus, one hypothesis for the limited geographic range of lineages 5 and 6 could be decreased fitness relative to strains better able to evolve antibiotic resistance. In this case, we might expect that mutations driving drug resistance in these two lineages would be different from those evolving in more successful lineages. Thus, we analyzed our newly sequenced strains from Mali for the presence of mutations known to confer drug resistance and used in common nucleic acid-based commercial tests [59, 60] for the detection of drug resistance [69–75] (S2A Table). Forty (60%) strains in lineages 1–4 and only four (15%) of the lineage 5 and 6 strains were phenotypically resistant to at least one of the four tested drugs. We observed that mutations used in commercial tests were sensitive in detecting phenotypic resistance to rifampicin, isoniazid and ethambutol (S2B Table and Figs 1 and S1).

Streptomycin resistance mutations were not included among our list of known resistance mutations (S2A Table). Therefore, we searched for potential resistance mutations in a set of genes previously known to affect streptomycin resistance, including *rrs*, *rpsL*, and *gidB* [76–78]. We identified a point mutation in *gidB* that caused a non-synonymous change (leucine to serine at residue 79) that is predicted to affect protein function [57] (S1D Fig; see S1 Text for more details). This mutation was found in 23 streptomycin resistant strains and no streptomycin susceptible strains in our dataset and likely represents a previously uncharacterized mutation that confers resistance to this drug. Previous studies have identified loss of function mutations in *gidB* affecting streptomycin resistance [77], as well as point mutations in the region of *gidB* close to residue 79, including at residues 75 and 82 [78].

In addition, we identified known mutations in genes associated with resistance to drugs that were not phenotypically assessed, including ofloxacin, kanamycin, and ethionamide. Using the list of mutations in S2A Table, we found that 25 (38%) of the Mali strains belonging to lineages 1, 2 or 4 could be classified as MDR (multi-drug resistant; resistant to isoniazid and rifampicin), and two (3%) could be classified as pre-XDR (pre-extensively drug resistant; resistant to isoniazid, rifampicin, plus either ofloxacin or kanamycin). In contrast, three (11%) of the lineage 5 and 6 strains could be classified as MDR, and one (4%) could be classified as pre-XDR. The presence of these pre-XDR strains is of particular concern, as XDR has not been reported in Mali, and testing is not currently performed routinely for second line antibiotics [79, 80].

Similar resistance-conferring mutations were found among the lineages (S2 Fig). Although we cannot eliminate the possibility of cross resistance and other alternate genetic mechanisms of lineage 5 and 6 drug resistance, or of differences in drug tolerance or rates of persister cells, it appears that the mechanism of genetic drug resistance was similar between lineages 2, 4, 5 and 6. Thus, although the sample size was small, our results suggest that drug resistance, while less frequent in lineage 6, evolves through acquisition of similar mutations as observed in lineages 2 and 4 in Mali, including combinations of mutations leading to pre-XDR, and that this resistance could be detected using current molecular diagnostic approaches.

### Individual lineage-specific features suggest additional mechanisms that could be involved in geographic restriction

Previous analyses pinpointing lineage 6-specific genomic features have compared limited numbers of strains, which might have caused these studies to miss important features or to identify features that are not actually found in a broader set of lineage 6 strains [8, 19]. Also, these studies have not examined genomes of lineage 5 in detail. Using both our Alignment and Assembly Collections, containing representatives from lineages 1 through 6 and *M*. *bovis*, we sought to robustly identify distinguishing features of lineages 5 and 6, focusing on traits that could have caused geographic restriction. Using our Assembly Collection, at each node labeled A-D in Fig 2 (representing genetic diversification events that may correlate with ecological specialization), we identified gene gains and losses (Table 1; Materials and Methods). Many of our findings agreed with previous observations describing regions of difference, as determined through genomic hybridizations [3, 18]. However, we also identified a small number of genes that were not previously identified as being part of these known regions of difference (see S1 Text for full details), including a gain of genes encoding a PE-PGRS and hypothetical protein at the last common ancestor of lineage 6 and *M*. *bovis*, the loss of *Rv1523* (a methyltransferase) and *Rv3514 (PE-PGRS57)* in lineage 5, and the loss of a gene encoding a TetR family regulator and the gain of one PPE protein-encoding gene at the last common ancestor of lineages 5, 6 and *M*. *bovis*.

**Table 1:** Orthologs identified in gene content analysis as lost or gained at nodes A-D. For some regions of difference (RD), the first and/or last gene in the region was not identified in our analysis because enough of the gene remained to align to H37Rv, and thus was not considered absent. Gene names starting with an O were identified through our SYNERGY orthogroup analysis. Nodes A-D were identified in Fig 2.

A: root node of lineage 6				C: root node of lineage 5			
	**loss**	**Annotation**	**RD**		**loss**	**Annotation**	**RD**
	Rv0124	PE-PGRS family protein PE_PGRS2	RD701		Rv1334	hydrolase	RD711
B: root node of lineage 6 and *M*. *bovis*					Rv1335	sulfur carrier protein CysO	RD711
	**loss**	**Annotation**	**RD**		Rv1523	methyltransferase	
	Rv0222	enoyl-CoA hydratase EchA1	RD10		Rv1978	hypothetical protein	RD713
	Rv1965	ABC transporter permease YrbE3B	RD7		Rv1979c	permease	RD713
	Rv1966	MCE-family protein Mce3A	RD7		Rv1993c	hypothetical protein	RD743
	Rv1967	MCE-family protein Mce3B	RD7		Rv1994c	ArsR family transcriptional regulator CmtR	RD743
	Rv1968	MCE-family protein Mce3C	RD7		Rv1995	hypothetical protein	RD743
	Rv1969	MCE-family protein Mce3D	RD7		Rv3514	PE-PGRS family protein PE_PGRS57	
	Rv1970	MCE-family lipoprotein LprM	RD7	D: root node of lineages 5 and 6 and *M*. *bovis*			
	Rv1971	MCE-family protein Mce3F	RD7		**loss**	**Annotation**	**RD**
	Rv1972	MCE-associated membrane protein	RD7		Rv2073c	oxidoreductase	RD9
	Rv1973	MCE-associated membrane protein	RD7	** **	Rv2074	pyridoxamine 5'-phosphate oxidase	RD9
	Rv1974	membrane protein	RD7		Rv2084	hypothetical protein	
	Rv1975	hypothetical protein	RD7				
	Rv1976c	hypothetical protein	RD7		**gained**	**Annotation**	**RD**
	Rv3617	epoxide hydrolase EphA	RD8		O850451604	PPE family protein	
	Rv3618	monooxygenase	RD8				
	Rv3619c	ESAT-6 like protein EsxV	RD8				
	Rv3620c	ESAT-6 like protein EsxW	RD8				
	Rv3621c	PPE family protein PPE65	RD8				
	Rv3622c	PE family protein PE32	RD8				
							
	**gained**	**Annotation**	**RD**				
	O850447630	hypothetical protein					
	O850450572	PE-PGRS family protein					

In addition, using our alignments to *M*. *tuberculosis* H37Rv, we identified a number of lineage-specific mutations, including pseudogenes that affect protein function (Tables 2, S4, S5 and S6). From these data, we identified 681 lineage 6-specific mutations shared across all lineage 6 strains, including eight truncated pseudogenes. These data also provided the first in-depth analysis of lineage 5 assemblies, which revealed 952 lineage-specific mutations and 43 pseudogenes as shared by our two lineage 5 strains (see S1 Text). The larger number for lineage 5 compared to other lineages likely results from our small sample size. Key categories of lineage-specific mutations and pseudogenes that might contribute to the geographic restriction of lineages 5 and 6 are discussed below, and in more detail in the S1 Text.

**Table 2:** Summary of the lineage-specific mutations and pseudogenes detected for each lineage.

	Mutations		Pseudogenes	
Lineage	Average ± SD	Lineage Specific	Average ± SD	Lineage Specific
LIN-1	2527.2 ± 39.2	536	189 ± 21.5	5
LIN-2	1774.4 ± 64.9	308	183.5 ± 43.1	23
LIN-3	1740.0 ± 52.9	406	157.9 ± 46.5	5
LIN-4	1050.0 ± 143.9	1	99.5 ± 28.8	0
LIN-5	2540.5 ± 4.9	952*	190.5 ± 0.7	43*
LIN-6	2605.8 ± 82.0	681	201.5 ± 26.4	8
LIN-5 and LIN-6	2600.4 ± 80.5	90	200.5 ± 25.4	5
LIN-1, LIN2, LIN-3, and LIN-4	1355.4 ± 400.0	0	148.4 ± 55.4	0
LIN-1, LIN2, and LIN-3	1803.4 ± 161.9	NA	182.3 ± 42.5	NA

*Due to low lineage 5 sample size (only two lineage 5 strains), there is low confidence in which mutations were lineage 5-specific.

### Mutations affecting ESX secretion could contribute to differing immune responses elicited by lineages 5 and 6

One distinguishing clinical characteristic of lineage 6 is an attenuated T cell response to ESAT-6, one of the proteins secreted through the ESX secretion system, as compared to patients infected with lineages 1–4 [5]. This altered immune response supports the hypothesis that lineage 5 and 6 have specificity for a particular host immunogenic background. Although our data cannot address whether ESAT-6 production has been affected, we observed non-synonymous polymorphisms, including indels, in genes encoding ESX secretion systems that could contribute to the different immune responses of lineage 6-infected patients (Table 3). Furthermore, we observed lineage-specific mutations in ESX-encoding genes in all lineages, suggesting that each lineage may have unique interactions with the host (Table 3; S1 Text).

**Table 3:** Summary of lineage-specific mutations highlighted in the results and discussion. Each cell indicates the number of lineage-specific mutations in that category (see Materials and Methods). “Indel” indicates an insertion or deletion; “synonymous” indicates a SNP that does not change the amino acid sequence; “non-synonymous” indicates a SNP that alters the amino acid sequence; and “non-synonymous and affects protein function” is the subset of non-synonymous SNPs that were predicted by SIFT [57] to affect protein function. Each column heading refers to the gene category, each of which is discussed in more detail in the results section. Due to low lineage 5 sample size (only two lineage 5 strains), there is low confidence in which mutations were lineage 5-specific.

	Category:	ESX Secretion	molybdenum, riboflavin and cobalamin	L,D transpeptidase	adenylate cyclase	drug-resistance associated genes	mammalian cell entry (MCE)
Lineage 1	Indel	0	1	0	0	0	0
	Synonymous	3	2	0	1	0	1
	Non-synonymous	4	3	0	1	1	1
	Non-synonymous & affects protein function	1	2	0	0	1	1
Lineage 2	Indel	1	1	0	0	0	0
	Synonymous	2	2	0	0	0	0
	Non-synonymous	7	0	0	0	0	0
	Non-synonymous & affects protein function	3	0	0	0	0	0
Lineage 3	Indel	0	0	0	0	0	0
	Synonymous	2	1	0	2	1	2
	Non-synonymous	3	0	0	0	1	1
	Non-synonymous & affects protein function	0	0	0	0	0	1
Lineage 5	Indel	3	1	0	0	0	1
	Synonymous	10	7	0	2	1	0
	Non-synonymous	16	8	1	2	2	6
	Non-synonymous & affects protein function	7	4	1	2	2	4
Lineage 6	Indel	2	0	0	1	0	0*
	Synonymous	1	7	0	0	0	1
	Non-synonymous	7	7	2	2	3	3
	Non-synonymous & affects protein function	1	3	1	0	0	1
Lineage 5 & 6	Indel	0	2	0	0	0	0
	Synonymous	2	0	0	0	0	0
	Non-synonymous	0	0	0	0	0	1
	Non-synonymous & affects protein function	0	0	0	0	0	1

*Table does not include the 8 genes completely deleted in RD7

### Alterations in cofactor biosynthesis pathways could impact many cellular functions in lineages 5 and 6

Lineages 5 and 6 had lineage-specific mutations, including pseudogenes, in genes encoding multiple components of cofactor biosynthetic pathways, including molybdenum, vitamin B12, and vitamin B3 (S1 Text and Tables 3, S4 and S5). Molybdenum cofactors are key catalysts for redox reactions, and are hypothesized to have played an important role in the evolution of pathogenic mycobacteria [81]. In addition, mycobacteria are one of the few bacterial pathogens with the ability to synthesize vitamin B12 [82]. Thus, both of these cofactors have specifically evolved in mycobacteria and loss of these cofactor biosynthetic pathways could affect the function of proteins that use these cofactors, which include proteins that are important for many cellular functions. These mutations may affect the host range of lineages 5 and 6, supporting the hypothesis of a unique host preference.

### Mutations that could cause distinct cell size, morphology and growth of lineages 5 and 6

It has been shown previously that *M*. *africanum* GM041182 has a distinct physiology as compared to that of *M*. *tuberculosis* H37Rv, including a larger cell size and slower growth rate [7]. Possibly explaining these differences, we identified lineage 6-specific non-synonymous SNPs in genes encoding the L,D transpeptidases, *ldtA* and *ldtB* (*Rv0166c* and *Rv2518c*), previously shown to form cross-linkages within peptidoglycan (Tables 3 and S4A) [83] and to be key drivers of cell shape, size, surface morphology, growth and virulence [84]. Lineage 5 also contained a non-synonymous SNP predicted to affect LdtA protein function (Tables 3 and S4A). No other lineages had a lineage-specific mutation in an L,D-transpeptidase.

### Mutations affecting cAMP signaling in lineages 5 and 6 could affect virulence

We observed that lineages 5 and 6 had lineage-specific mutations in genes encoding adenylate cyclases, the enzymes that synthesize cyclic AMP (cAMP), an important cell signaling molecule. Although the affected genes were different between the two lineages, no other lineage had lineage-specific mutations predicted to affect adenylate cyclase function. Deletion of one of the 17 adenylate cyclases in *M*. *tuberculosis*, *Rv0386*, has been shown to reduce virulence and alter the immune response [85]. Bentley et al. also previously found that this gene was a pseudogene in *M*. *africanum* GM041182, although here we find that pseudogenization of *Rv0386* was not lineage specific (S6A Table). Nevertheless, given the number of affected adenylate cyclases, there may be differences in cAMP signaling within lineages 5 and 6, leading to altered pathogenicity.

### Distinct mutations in drug resistance-associated genes in lineages 5 and 6

In order to shed light on the reported lower rates of drug resistance in lineages 5 and 6, we screened our lineage-specific mutations to investigate if there were any changes in known drug resistance genes that were not on the list of mutations used before and that might affect the development of antibiotic resistance [20, 68]. In lineage 6, we observed two lineage-specific non-synonymous mutations in *rpoB*, and one lineage-specific non-synonymous mutation in *embC* (S1A and S1C Fig and Tables 3 and S4A) not previously implicated in antibiotic resistance. Lineage 5 strains had non-synonymous mutations in genes encoding AtpH (*Rv1307*) and AtpG (*Rv1309*), both of which are subunits of ATP synthase [86] (S4A Table), and a target of bedaquiline, a new antibiotic reserved for the treatment of drug resistant tuberculosis [87]. Both of these mutations were predicted to affect protein function by SIFT [57], and may affect bedaquiline efficacy in countries with a high proportion of patients infected with lineage 5. Thus, both of these lineages have non-resistance conferring mutations in genes associated with drug resistance that might influence the frequency at which drug resistance develops in these lineages.

### Mutations in mammalian cell entry genes that could affect virulence in lineages 5 and 6

*M*. *tuberculosis* H37Rv contains four mammalian cell entry (MCE) operons, which play an important role in mycobacterial virulence [88]. In addition to confirming earlier reports that lineage 6 strains lacked one of these four operons (operon 3; Table 1) [18], we observed lineage-specific mutations in several of the other MCE operons (lineage 6 had mutations in operons 1 and 2; lineage 5 had mutations in operons 1 and 3). We also observed a non-synonymous mutation in *mce1B* that was shared by lineages 5 and 6 strains and was predicted by SIFT to affect Mce1B protein function [57]. In comparison, the other lineages had nearly identical MCE operons as compared to *M*. *tuberculosis* H37Rv (Tables 3 and S4A).

## Discussion

Our study describes the largest collection of sequenced lineage 6 isolates to date, and, to our knowledge, the first in-depth analysis of the genetics of lineage 5. Through our work, we have characterized the genetic basis of antibiotic resistance in lineage 6 strains from Mali, shown that *M*. *africanum* and *M*. *tuberculosis* are part of the same species, and better defined the mutations and changes in gene content that typify these lineages. Collectively, this work provides insights into these understudied lineages and provides testable hypotheses as to why they are geographically restricted.

We evaluated 92 Mali MTC isolates using both assembly and alignment-based approaches. Our assemblies revealed several new regions of difference and our alignments identified smaller lineage-specific changes. In addition to our conclusion that *M*. *africanum* is not a separate species, we observed that some *M*. *africanum-M*. *tuberculosis* pairs of strains have greater average nucleotide identity than some pairs of strains from within the same lineage. Furthermore, our ANI data demonstrated that there is comparable diversity in lineages 4 and 6, suggesting that lineage 6 has not undergone a recent population bottleneck. This emphasizes the extremely close relationship between all MTC lineages, highlighting the role that small changes within the MTC have played in geographical restriction and altering host preferences. Since our assemblies were of very high quality, we were able to observe changes in genes that previous studies could not, thus providing a prioritized list of genes for investigating lineage 5 and 6 characteristics.

One hypothesis for the geographical restriction of lineages 5 and 6 is the presence of an unknown non-human reservoir. *M*. *africanum* has been found in animals, including monkeys, cows, pigs and hyrax [89–94]. Unfortunately, given genomic data from human clinical isolates alone, we cannot address this hypothesis directly. However, given the similar level of diversity between lineage 4 and 6 in Mali and the evidence of person-to-person transmission, a non-human reservoir seems unlikely to explain the geographic restriction, as lineage 6 appears well adapted to spread in humans living in this geographic setting, unlike *M*. *bovis* in this and other settings [95–97].

Another hypothesis for why lineages 5 and 6 occur almost exclusively in West Africa is a preference for hosts of West African ethnicity, supported by previous evidence, including a study linking lineage 5 to the Ewe ethnic group [17]. We identified lineage-specific mutations in ESX genes in every lineage, indicating that each lineage may interact uniquely with the host immune system. Mycobacteria have five ESX secretion systems, also known as type VII secretion systems, which secrete small proteins across the bacterial cell envelope and are important to mycobacterial virulence [98, 99]. For example, ESX-1 secretion is lost as part of RD1 in *M*. *bovis* BCG vaccine strains, resulting in loss of ESAT-6 and CFP-10 secretion, and thus attenuation of the bacterium [100, 101]. The lineage-specific mutations in ESX genes could lead to alterations in the pathogen-host immune interaction, resulting in a requirement in lineages 5 and 6 for the West African immune system. In fact, an altered response to ESAT-6 in patients infected by lineage 6 has previously been reported [5]. Thus, the specific ESX mutations in lineages 5 and 6 could represent adaptations to the niche of the West African host.

Lineage-specific mutations in cobalamin biosynthesis could also contribute to adaptation of these lineages to the specific ecological niche of the West African host. The hypothesis of adaptation to a different host cofactor environment for lineages 5 and 6 is supported by several studies that have found increased levels of vitamin B12 plasma concentrations in West Africans compared to Europeans and Mexicans [102, 103]. One unique characteristic of mycobacteria compared to many other bacterial genera is that they are capable of synthesizing vitamin B12. Furthermore, vitamin B12 may play a crucial role in *M*. *tuberculosis* infection [82, 104, 105]. Thus, the lineage-specific mutations in cobalamin pathways in lineages 5 and 6 may alter these strains’ ability to synthesize vitamin B12, which may be tolerated in West African hosts with higher levels of plasma B12. Adaptation to this B12-rich West African niche might prevent these lineages from infecting other ethnic groups with lower B12 bioavailability; however, further studies would be required to confirm this hypothesis.

A third hypothesis for the geographic restriction of lineages 5 and 6 is that they are less fit, either for transmission or in-host virulence, resulting in a decreased ability to survive outside of West Africa. Several papers have shown no difference in transmission rates between *M*. *tuberculosis*-associated strains and *M*. *africanum*-associated strains [5, 9, 106, 107]. In agreement with previous findings, our Mali Collection revealed three pairs of lineage 6 strains separated by 10 or fewer SNPs when aligned to *M*. *tuberculosis* H37Rv, suggesting recent transmission of strains between patients within the ethnic backgrounds prevalent in Mali [64]. That these transmission events were not exclusive to HIV positive patients suggests that a compromised immune system is not required for a transmission event. These results indicate that lineages 5 and 6 do not have a reduced ability to transmit.

A decrease in fitness could also be reflected in a decrease in virulence. It has been hypothesized that *M*. *africanum* is less virulent within humans, mice and guinea pigs than is *M*. *tuberculosis* [7, 9–11]. However, lineage 6 was not enriched for mutations in virulence and growth-related genes compared to lineages 1, 2 and 3, suggesting that lineage 6 does not contain an overall numerical loss of virulence or growth-associated genes. Despite this, individual mutations can still greatly affect disease outcome, and analysis of lineage-specific mutations identified several potential mechanisms that could lead to changes in how lineages 5 and 6 proliferate and cause disease. The lineage-specific mutations discussed above that could relate to a niche adaptation in hosts of West African ethnicity, including the lineage-specific mutations in ESX genes and cofactor biosynthesis genes, are also involved in virulence.

Another key set of virulence genes with lineage 5 and 6-specific mutations are the MCE operons. The MCE operons play an important role in the virulence of *M*. *tuberculosis*, particularly in mycobacterial growth in macrophages [67]. Antibodies to MCE1 proteins have been identified in patients [108], and operons 1–3 are required for virulence in mice [88]. Despite this apparent role in virulence, lineage 6 contains mutations that affect protein function in operons 1–3, while lineages 1–3 have nearly identical MCE operons to *M*. *tuberculosis* H37Rv, suggesting one potential mechanism of decreased virulence.

Another set of virulence-related genes with lineage-specific mutations are adenylate cyclases, which synthesize cAMP, an important second messenger [109]. *M*. *tuberculosis* encodes 17 adenylate cyclases, and deletion of one of them (*Rv0386*) has been shown to affect virulence and host response [85], highlighting the importance of this set of genes to pathogenicity. Both lineages 5 and 6 contained lineage-specific mutations predicted to affect the protein function of several adenylate cyclases, suggesting altered cAMP signaling in these strains, and a potential effect on the virulence of lineages 5 and 6.

Another pathway that affects bacterial growth and host response is the synthesis of the cell wall. Both lineage 5 and 6 contained lineage-specific mutations in L,D-transpeptidases. L,D-transpeptidases are critical to the structure of mycobacterial peptidoglycan and are involved in bacterial structure and growth [84], providing a possible explanation for the reported changes in cell size and doubling time in *M*. *africanum* GM041182 compared to *M*. *tuberculosis* H37Rv [7]. An altered cell wall could support either the hypothesis of decreased virulence, or suggest the need for a specific host immune system.

In addition, we saw high variability in PE, PPE and PE-PGRS genes, including changes in gene content. These repetitive regions are difficult to sequence and are often ignored, but may play a crucial role in antigenicity and the host-pathogen interaction [110, 111]. Using our high quality assemblies and alignments, we were able to identify lineage-specific mutations in these genes, as well as altered gene content. These mutations highlight the possibility of a critical role for these proteins in host-pathogen interactions and emphasize the need for a more detailed analysis of these regions. Furthermore, there were also a number of mutated hypothetical proteins and proteins of unknown function, all of which may play a critical as yet undiscovered role.

In addition to exploring mechanisms of geographic restriction, we also identified mutations that may have clinical implications for the region. We found that in Mali, *M*. *africanum*-associated and *M*. *tuberculosis*-associated strains evolved antibiotic resistance through similar mutations, and thus standard line-probe assays can still be utilized in West Africa. However, we also found a *gidB* polymorphism not previously described which might account for much of the streptomycin resistance in Mali. Also of concern, we identified several cases of pre-XDR in Mali, suggesting that Mali may need to begin testing for XDR cases. Furthermore, we identified lineage 5 or 6-specific mutations that may affect the evolution of drug resistance, particularly bedaquiline. Thus, whole genome sequencing surveys like this one are useful in revealing new mechanisms for drug resistance, informing development of molecular diagnostics.

One weakness of our study was that we were limited in our sample size for lineage 5 and *M*. *bovis* strains. Our collection was not representative of *M*. *bovis* genomic diversity, as three of the four *M*. *bovis* strains in our analysis were attenuated *M*. *bovis BCG* vaccine strains. However, we only used the *M*. *bovis* strains in our ANI and gene content analysis, and required that any observations be consistent with wild-type *M*. *bovis* sequence, AF2122/97, and our results corroborated all previous findings of *M*. *bovis* regions of difference. Another weakness of our study was that our observations may be specific to Mali, since all lineage 5 and 6 isolates sequenced for our study were isolated in Mali, although these lineages are found throughout West Africa. However, our lineage 6 isolates were genetically diverse, and represented multiple spoligotypes, and our isolates from other lineages did not cluster separately on the phylogenetic tree from strains isolated from South African patients. Thus, our collection reflected substantial diversity and did not originate from a clonal outbreak. In fact, the study from which we selected our samples found a wide diversity of strains in Mali, which covered 55% of all known spoligotyped strains [20]. Furthermore, based on spoligotyping, many similar strains can be found in neighboring countries [54, 68, 112–115]. And, finally, studies that employ genomic data alone are insufficient to address causality. However, we believe that this in-depth genomics analysis of the neglected pathogen, “*M*. *africanum*”, provides a strong foundation from which causal relationships between lineage-specific variation and geographic restriction can be made.

This collection provides valuable insights into the distinguishing genomic features of *M*. *africanum*. Here, we have analyzed in detail the genomes of lineage 5 and 6 isolates from Mali and identified several potential genetic reasons for the geographical restriction of lineages 5 and 6, such as alterations in vitamin B12 pathways and genes associated with virulence, which provide a guide to future studies focusing on the effects of specific genes. Although we cannot specifically point to a single reason why these lineages are geographically restricted, we have found mutations that support the hypothesis of a unique requirement for a host of West African ethnicity and for the hypothesis of loss of bacterial fitness. These hypotheses are not mutually exclusive, and we anticipate that these observations will be able to inform and fast-track experiments on mycobacterial pathogenicity and virulence, particularly with regard to this unique member of the MTC.

## Supporting Information

S1 Fig Comparison of our Mali collection to the previously observed distribution of West African spoligotypes. Percent of strains in our Mali collection (black bars) or in West Africa (gray bars) based on (A) the spoligotype international type (SIT) or (B) spoligotype clade. West African numbers are based on the SITVITWEB database [45]. SITs or clades present at less than 1% in either group were excluded from the figure.
(PDF)

S2 Fig Mutations in drug-resistance associated genes. Plots showing details of mutations identified in genes known to confer drug resistance. Light blue or red horizontal shaded bars indicate phenotypic sensitivity or phenotypic resistance, respectively, for the strain of interest. The corresponding vivid color in a particular box indicates the presence of the resistance mutation represented by that column. A) rifampicin B) isoniazid C) ethambutol D) streptomycin.
(PDF)

S1 Table Samples used in our study. A) List of Mali Collection samples, with patient information and digital spoligotype results. B) List of all 137 Assembly Collection strains, used for our assembly-based analyses, including 91 newly sequenced strains from Mali, 40 strains from South Africa [25], and six strains from Genbank. C) Sequence Read Archive identifiers for each of the 161 additional strains from China used in our SNP analysis as part of the Alignment Collection [27].
(XLSX)

S2 Table Drug resistance analysis. A) Drug resistance mutations analyzed. B) Table showing true positives, false positives, true negatives, false negatives, sensitivity, and specificity for the four drugs for which we have phenotype information for all strains.
(XLSX)

S3 Table Schematic overview of key metrics for lineage specificity calculations. (DOCX)

S4 Table Lineage-specific mutations. All lineage-specific mutations (A) in coding sequences and (B) in intergenic regions. Maf indicates mutations shared between lineages 5–6, but not found in lineages 1–4. No mutations were shared between lineages 1–4 but not lineages 5–6.
(XLSX)

S5 Table Lineage-specific pseudogenes. Maf indicates mutations shared between lineage 5 and 6 but not found in lineages 1–4. No mutations were shared between lineages 1–4 but not lineages 5–6.
(XLSX)

S6 Table Comparison of pseudogenes to previous analysis. A) Comparison of pseudogenes identified differently by our study to those identified by Bentley et al [19]. This table compares lineage 4, lineage 6 or *M*. *africanum*-specific pseudogenes identified in our study to pseudogenes identified by Bentley et al. as belonging to lineage 4, lineage 6, lineage 6 and animal strains, or lineages 5–6 and animal strains. A “0” indicates that the gene is not a pseudogene in that strain, while “1” indicates that it is, and “2” indicates an ambiguous call. Genes with a light blue background were identified in this study and not by Bentley et al., while genes with a light green background were identified by Bentley et al., but not by this study, and genes with a purple background were identified by both studies. B) Table summarizing the differences between our study and Bentley et al. [19].
(XLSX)

S1 Text Additional information on the novel streptomycin resistance mutation and on the lineage-specific mutations. (DOCX)
