# Supplementary figures and images for: Whole Genome Sequencing of Mycobacterium africanum Strains from Mali Provides Insights into the Mechanisms of Geographic Restriction
Source: PLoS Negl Trop Dis. 2016 Jan 11;10(1):e0004332. doi: 10.1371/journal.pntd.0004332 (PMC4713829; doi:10.1371/journal.pntd.0004332)

**A**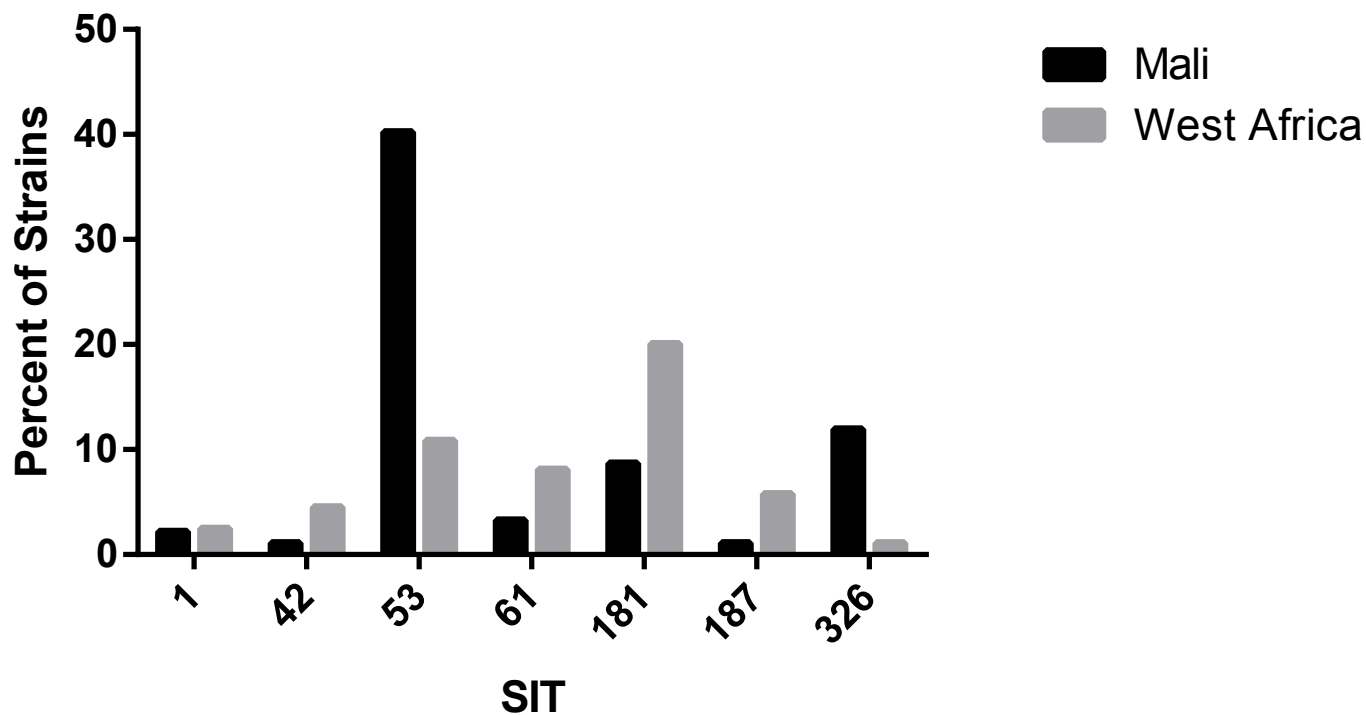**B**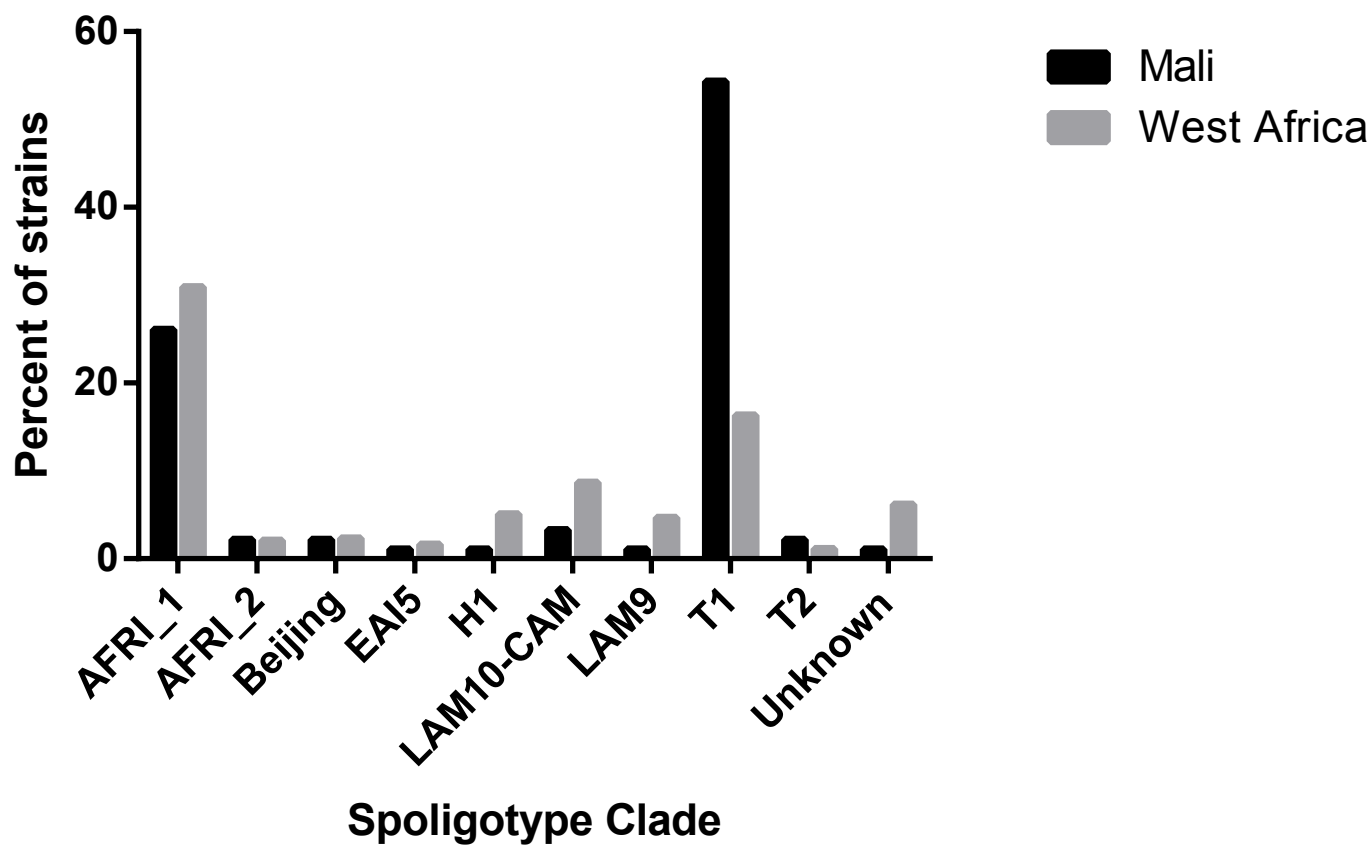

Supplement: S1 Fig — Percent of strains in our Mali collection (black bars) or in West Africa (gray bars) based on (A) the spoligotype international type (SIT) or (B) spoligotype clade. West African numbers are based on the SITVITWEB database [45]. SITs or clades present at less than 1% in either group were excluded from the figure. (PDF) [file pntd.0004332.s001.pdf]

Arifampicin  
mgit rmp 10ugml

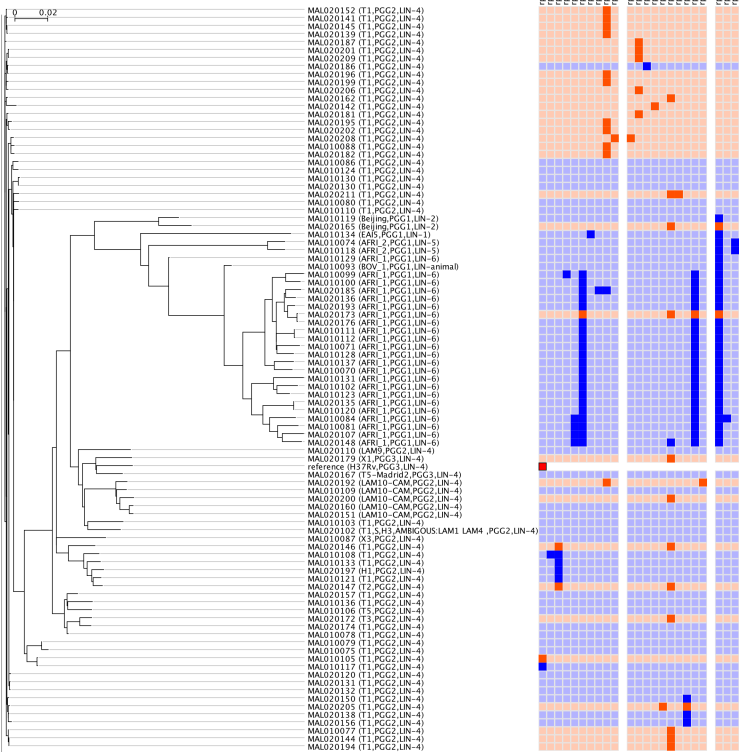

B  
isoniazid  
mgit inh low 01 ugm

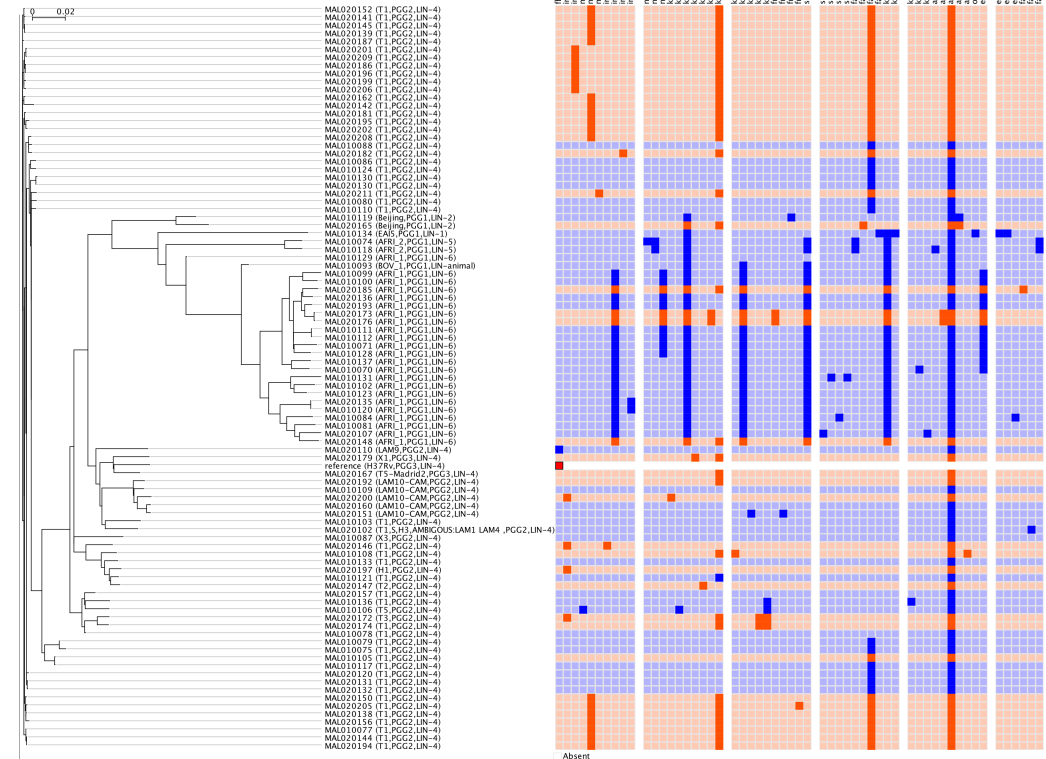

☐ Absent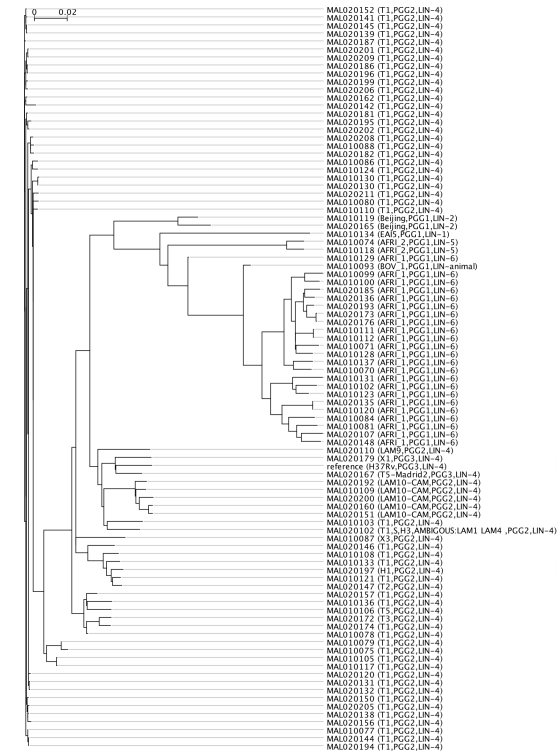

Dstreptomycin  
mgit sm 08ugml

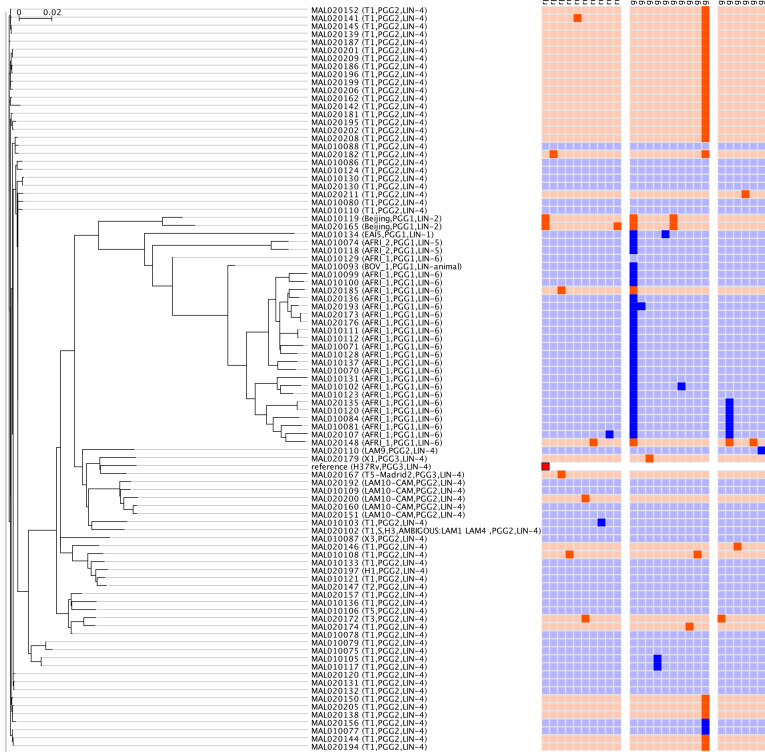

Supplement: S2 Fig — Plots showing details of mutations identified in genes known to confer drug resistance. Light blue or red horizontal shaded bars indicate phenotypic sensitivity or phenotypic resistance, respectively, for the strain of interest. The corresponding vivid color in a particular box indicates the presence of the resistance mutation represented by that column. A) rifampicin B) isoniazid C) ethambutol D) streptomycin. (PDF) [file pntd.0004332.s002.pdf]
