# Supplementary material for: Whole Genome Sequencing of Mycobacterium africanum Strains from Mali Provides Insights into the Mechanisms of Geographic Restriction
Source: PLoS Negl Trop Dis. 2016 Jan 11;10(1):e0004332. doi: 10.1371/journal.pntd.0004332 (PMC4713829; doi:10.1371/journal.pntd.0004332)
Supplement: S3 Table — (DOCX) [file pntd.0004332.s005.docx]

|  | Variant Present | Variant Absent |  |
| --- | --- | --- | --- |
| Lineage X | True positive (TP) | False negative (FN) | **Positive predictive value:** $PPV=\frac{TP}{TP+FN}$ |
| Lineage Other Than X | False positive (FP) | True negative (TN) | **Negative predictive value:** $NPV=\frac{TN}{FP+TN}$ |
|  | **True positive rate:** $TPR=\frac{TP}{TP+FP}$ | **True negative rate:** $TNR=\frac{TN}{TN+FN}$ |  |
