# Supplementary material for: Whole Genome Sequencing of Mycobacterium africanum Strains from Mali Provides Insights into the Mechanisms of Geographic Restriction
Source: PLoS Negl Trop Dis. 2016 Jan 11;10(1):e0004332. doi: 10.1371/journal.pntd.0004332 (PMC4713829; doi:10.1371/journal.pntd.0004332)
Supplement: S1 Text — (DOCX) [file pntd.0004332.s009.docx]

# Supplemental Text

## Novel drug resistance mutation in *gidB*

When we looked at mutations in genes known to play a role in resistance to streptomycin (*rrs*, *rpsL*, and *gidB*) [[1-3](#_ENREF_1)], we observed that 23 streptomycin resistant strains harbored a non-synonymous point mutation in the *gidB* gene (S2d Fig.). While SNPs in the *gidB* gene have previously been reported in association with streptomycin resistance, this particular SNP has not yet been identified [[2](#_ENREF_2), [3](#_ENREF_3)]. This mutation, at nucleotide position 236, changes a leucine to a serine, and was predicted by the SIFT algorithm [[4](#_ENREF_4)] to affect *gidB* protein function. Of the remaining 12 strains with unexplained streptomycin mutation, 10 had different mutations in *gidB*, and 2 had mutations in *rpsL*, another gene known to have a role in resistance to streptomycin [[5](#_ENREF_5)]. Thus, it appears that strains circulating in Mali might have evolved streptomycin resistance primarily via a previously undescribed change in *gidB*.

## Evolutionary history: Nodes A-D

At each node we used a combination of assembly and alignment data to identify distinguishing characteristics of the putative ancestral strain at that node. First, we used the assembly collection to identify orthologs that were either gained or lost at each node (Table 1; Materials and Methods). Many of these genes fell into already known regions of difference (RDs), previously identified by genome hybridizations [[6](#_ENREF_6), [7](#_ENREF_7)]. For some RDs, the first and/or last gene in the region was not identified in our analysis because enough of the gene remained to align to *M. tuberculosis* H37Rv, and thus was not considered absent. Second, we used functional Pfam annotation of genes from the assembly collection to detect protein domains that were significantly enriched or reduced within the members of each group. Third, we used variant calls from our alignment collection to identify other mutations (smaller than a gene) that were enriched or specific to each group, including those that caused truncations of genes (pseudogenes) that are likely to affect protein function (S4, S5 and S6 Tables). Our alignment collection enabled us to more accurately determine what features were specific to a particular lineage, and showed that many mutations and pseudogenes previously identified as lineage-specific were not lineage-specific when evaluating our larger set of strains (S6 Table).

### Node A: root node of lineage 6

The Mali collection provided a large and diverse collection of lineage 6 strains, which allowed us to more fully characterize the genetic content of this lineage than had been done previously [[8](#_ENREF_8), [9](#_ENREF_9)]. We observed no gene gains at the root node of lineage 6 (Node A in Fig. 2), and only one gene loss (previously identified as part of RD701), as expected from earlier phylogenetic work (Table 1) [[7](#_ENREF_7)].

When reads from lineage 6 strains were aligned to *M. tuberculosis* H37Rv, we observed 681 lineage 6-specific mutations, 82 of which were in intergenic regions and the rest (599) were within coding sequences, including 8 that resulted in truncated proteins likely to have abolished function (pseudogenes) (Table 2, S4, S5). Five of these pseudogenes had previously been identified, while three were novel (S6 Table) [[8](#_ENREF_8)]. S6a Table provides a side-by-side perspective of the lineage 6 pseudogenes that we detected and demonstrates that the genes identified by Bentley et al., but not by this study, were either pseudogenes in some other lineages, or were not pseudogenes in all lineage 6 strains.

### Node B: root node of lineage 6 and *M. bovis*

*M. africanum* was first identified due to its intermediate phenotype between *M. bovis* and *M. tuberculosis*, which was confirmed by its genetic position on the MTC tree [[10](#_ENREF_10), [11](#_ENREF_11)]. Based on this tree, and the tree generated by our own data, lineage 6 and *M. bovis* share a root ancestor separate from all other MTC lineages (Node B in Fig. 2). Thus, although we did not have alignment data for *M. bovis,* we were interested in how the genetic content of these two lineages compared. *M. bovis* and lineage 6 shared loss of RD10, RD7, and RD8 compared to lineage 5 (Table 1), corresponding with previous observations [[6](#_ENREF_6)]. Our Pfam analysis revealed that these two lineages also share a copy number reduction in the MCE Pfam category (PF02470.15), as a result of loss of MCE operon 3, which is contained in RD7. In addition, we identified two genes that were gained at this node (Table 1). One of these genes is a PE-PGRS protein; the other is a hypothetical protein.

### Node C: root node of lineage 5

Though our dataset contained only two lineage 5 strains, they provided valuable insights into the specific features of this lineage. For example, our gene content analysis revealed the loss of RD711, RD713, and RD743 at Node C, as expected, but also identified two additional lineage specific gene losses that were not part of any known region of difference [[7](#_ENREF_7)] (Table 1). One of these genes is *Rv1523*, which is annotated as a methyltransferase, and is most likely S-adenosyl-L-methionine dependent. KEGG predicts this gene to be in the pathways for tyrosine metabolism (ko00350) and polycyclic aromatic hydrocarbon degradation (ko00624). The other novel lost gene is *Rv3514*, annotated as PE-PGRS67. This is part of a larger family of proteins that are highly polymorphic and may play a role in antigenicity.

In addition to these larger changes, we identified 952 lineage 5-specific mutations and 43 lineage 5- specific pseudogenes (Table 2, S4, S5). The larger number of lineage-specific mutations and pseudogenes compared to the other lineages was a result of our small sample size, which required us to use different cutoffs. However, the average number of mutations per strain for lineage 5 was comparable to the other lineages in our study (Table 2).

### Node D: root node of lineage 5, 6 and *M. bovis*

Through genomic hybridization studies, it is known that all *M. bovis* and *M. africanum* strains share the loss of RD9 [[6](#_ENREF_6)]. Our gene content analysis of Node D confirmed this finding and identified one additional gene lost at this node and one gained gene (Table 1). The lost gene was *Rv2084*, which is annotated as a hypothetical protein but had BLAST similarity to a TetR family transcriptional regulator. The gained gene was a PPE family protein. In addition, our analysis of changes in Pfam content revealed loss of two families. One of these was PF13276.1, which consists of proteins with a helix-turn-helix domain. The helix-turn-helix is a motif involved in DNA binding. This suggested a change in gene expression in these three lineages, a suggestion supported by the fact that the other Pfam domain lost at this node was PF12349.3, a sterol-sensing Pfam domain. Since we did not have alignments of any *M. bovis* strains, we focused on mutations shared between lineages 5 and 6. There were 90 shared mutations, including 5 pseudogenes (S4, S5 Tables).

## Individual lineage-specific features suggest additional mechanisms that could be involved in geographic restriction

**Mutations affecting ESX secretion could contribute to differing immune responses elicited by lineages 5 and 6.** One distinguishing clinical characteristic of lineage 6 is an attenuated T cell response to ESAT-6 in patients infected with this lineage as compared to patients infected with lineages 1-4 [[12](#_ENREF_12)]. This altered immune response supports the hypothesis that there is lineage 5 and 6 specificity for a particular host immunogenic background. While it was hypothesized that the attenuated immune response was due to mutation of *Rv3879c*, which is part of the ESX-1 secretion pathway, complementation of this gene did not restore ESAT-6 secretion or presentation to T cells, suggesting that defective *Rv3879c* is not the cause of the altered immune response [[13](#_ENREF_13)]. Although Bentley et al. reported that *Rv3879c* was a pseudogene in *M. africanum* GM041182, we found that the pseudogenization of *Rv3879c* was not lineage specific, as not all lineage 6 strains encoded truncated versions of this protein, and many strains in other lineages were also truncated (S6a Table)[[8](#_ENREF_8), [13](#_ENREF_13)]. This suggested that inactivation of *Rv3879c* is unlikely to explain either the host preference or the altered host immune response of lineages 5 and 6. In fact, *Rv3879c* had lineage specific mutations in lineages 1, 2 and 5, but not lineage 6 (Table 3, S4a). However, we did observe lineage 5 and 6 specific polymorphisms in other genes involved in ESX secretion systems that might explain the different immune responses of lineage 6*-*infected patients as compared to those infected with other lineages (Table 3). Eight of the non-synonymous mutations were predicted to affect protein function, but even mutations not predicted to affect protein function might also affect the antigenicity of these secretion systems [[4](#_ENREF_4)]. In fact, we observed lineage specific mutations in ESX-encoding genes in all lineages, suggesting that each lineage may have unique interactions with the host (Table 3). Thus, our data show that ESX-secretion system polymorphisms are common across all MTC lineages and are not unique to lineages 5 and 6.

### Alterations in cofactor biosynthesis pathways could impact many cellular functions in lineages 5 and 6. Lineage 6 had lineage-specific mutations, including pseudogenes, in multiple components of important biosynthetic pathways, such as molybdenum uptake and cobalamin synthesis (Tables 3, S4, S5). Molybdenum containing enzymes are key catalysts for redox reactions, and are an important part of the evolution of pathogenic mycobacteria [[14](#_ENREF_14)]. We detected lineage 6-specific non-synonymous SNPs in two molybdopterin biosynthesis proteins and in the gene encoding the molybdenum transporter, ModC (Tables 3, S4).

In addition, mycobacteria are one of the few bacterial pathogens with the ability to synthesize vitamin B12, another important cofactor [[15](#_ENREF_15)]. Bentley et al. reported that two of the genes encoding the biosynthesis proteins, CobL and CobK, are pseudogenes in all lineage 5 and 6 strains [[8](#_ENREF_8)]. We found that loss of function in *cobL* is specific to both lineage 5 and 6 but *cobK*, while a pseudogene in both lineages, is also a pseudogene in all lineage 1 strains and some lineage 2 and lineage 4 strains (Tables 3, S5, S6). We also observed a lineage 6 specific non-synonymous SNP in c*obD*, although SIFT predicted that this change would not affect protein function (Table S4a) [[4](#_ENREF_4)]. Loss of these cofactor biosynthetic pathways could have ramifications on the function of proteins that use these cofactors, and thus could have indirect effects on host-pathogen interactions.

The vitamin B12 pathway was also mutated in lineage 5. Lineage 5 had a non-synonymous mutation in four of the cobalamin synthesis enzymes and two flavoproteins. In addition, there was a non-synonymous mutation in a riboflavin biosynthesis protein, part of the vitamin B3 biosynthetic pathway (Tables 3, S4a). The mutations in *cobO, cobM* and *cobU*, all important parts of the vitamin B12 pathway, were all predicted to affect protein function [[4](#_ENREF_4)]. Similarly, one of the molybdenum cofactor biosynthesis genes was a pseudogene while another contained a non-synonymous mutation (Table 3, S4a, S5). Besides the pseudogene in *cobK* in lineage 1 and some lineage 2 and 4 strains, lineage 2 had an insertion in *cobB* resulting in a pseudogene, while lineage 1 had non-synonymous mutations in three molybdenum-associated genes (Table S5). Likewise, only lineage 1 had a non-synonymous mutation (a deletion) in a riboflavin-associated gene (Table 3, S4a). Thus, though mutations in these genes are seen in other MTC lineages, lineages 5 and 6 seem to have an increased number of mutations in the vitamin B3 and B12 biosynthesis pathways. Differences in cofactor synthesis could indicate a lineage 5 and 6 preference for a different host environment, as in the case of an animal reservoir or variation in host immune pressures, particularly because West Africans have higher levels of B12 in plasma compared to Europeans [[16](#_ENREF_16), [17](#_ENREF_17)].

**Alterations in abundantly secreted proteins in lineages 5 and 6.** We also observed lineage 6 specific polymorphisms in other genes that are predicted to have a role in modulating the host’s immune response. For example, we identified non-synonymous changes in the gene encoding antigen 85B (*Rv1866*), a secreted immunogenic protein that has been proposed as a potential vaccine target (Table 3, S4a) [[18](#_ENREF_18)]. No other lineages had a lineage-specific mutation in this gene. Though SIFT predicted that this change would not affect protein function, NetMHCII, an online tool that predicts binding of peptides to MHC class II alleles, predicted that that this mutation would change the binding of one of the putative strong binding peptides [[4](#_ENREF_4), [19](#_ENREF_19)]. Although lineage 5 did not contain lineage-specific mutations in antigen 85B, we detected a lineage 5 specific SNP in another abundantly secreted protein, MPT64 (*Rv1980c)* (Table 3, S4a). This mutation was predicted to affect both protein function and binding to MHC class II molecules [[4](#_ENREF_4), [19](#_ENREF_19)]. Thus, part of the reason for geographical restriction of lineages 5 and 6 may be due to alterations in the pathogen-host immune interaction. This is a particular concern for vaccine development in West Africa.

## References

1. Finken M, Kirschner P, Meier A, Wrede A, Bottger EC. Molecular basis of streptomycin resistance in *Mycobacterium tuberculosis*: alterations of the ribosomal protein S12 gene and point mutations within a functional 16S ribosomal RNA pseudoknot. Mol Microbiol. 1993;9(6):1239-46. PubMed PMID: 7934937.

2. Wong SY, Lee JS, Kwak HK, Via LE, Boshoff HI, Barry CE, 3rd. Mutations in *gidB* confer low-level streptomycin resistance in *Mycobacterium tuberculosis*. Antimicrob Agents Chemother. 2011;55(6):2515-22. doi: 10.1128/AAC.01814-10. PubMed PMID: 21444711; PubMed Central PMCID: PMC3101441.

3. Farooqi JQ, Khan E, Alam SM, Ali A, Hasan Z, Hasan R. Line probe assay for detection of rifampicin and isoniazid resistant tuberculosis in Pakistan. J Pak Med Assoc. 2012;62(8):767-72. PubMed PMID: 23862246.

4. Kumar P, Henikoff S, Ng PC. Predicting the effects of coding non-synonymous variants on protein function using the SIFT algorithm. Nat Protoc. 2009;4(7):1073-81. doi: 10.1038/nprot.2009.86. PubMed PMID: 19561590.

5. Okamoto S, Tamaru A, Nakajima C, Nishimura K, Tanaka Y, Tokuyama S, et al. Loss of a conserved 7-methylguanosine modification in 16S rRNA confers low-level streptomycin resistance in bacteria. Mol Microbiol. 2007;63(4):1096-106. doi: 10.1111/j.1365-2958.2006.05585.x. PubMed PMID: 17238915.

6. Brosch R, Gordon SV, Marmiesse M, Brodin P, Buchrieser C, Eiglmeier K, et al. A new evolutionary scenario for the *Mycobacterium tuberculosis* complex. Proc Natl Acad Sci U S A. 2002;99(6):3684-9. doi: 10.1073/pnas.052548299. PubMed PMID: 11891304; PubMed Central PMCID: PMC122584.

7. Mostowy S, Onipede A, Gagneux S, Niemann S, Kremer K, Desmond EP, et al. Genomic analysis distinguishes *Mycobacterium africanum*. J Clin Microbiol. 2004;42(8):3594-9. doi: 10.1128/JCM.42.8.3594-3599.2004. PubMed PMID: 15297503; PubMed Central PMCID: PMC497617.

8. Bentley SD, Comas I, Bryant JM, Walker D, Smith NH, Harris SR, et al. The genome of *Mycobacterium africanum* West African 2 reveals a lineage-specific locus and genome erosion common to the *M. tuberculosis* complex. PLoS Negl Trop Dis. 2012;6(2):e1552. doi: 10.1371/journal.pntd.0001552. PubMed PMID: 22389744; PubMed Central PMCID: PMC3289620.

9. Gehre F, Otu J, DeRiemer K, de Sessions PF, Hibberd ML, Mulders W, et al. Deciphering the growth behaviour of *Mycobacterium africanum*. PLoS Negl Trop Dis. 2013;7(5):e2220. doi: 10.1371/journal.pntd.0002220. PubMed PMID: 23696911; PubMed Central PMCID: PMC3656116.

10. Castets M, Boisvert H, Grumbach F, Brunel M, Rist N. [Tuberculosis bacilli of the African type: preliminary note]. Rev Tuberc Pneumol (Paris). 1968;32(2):179-84. PubMed PMID: 4985104.

11. Coscolla M, Gagneux S. Consequences of genomic diversity in *Mycobacterium tuberculosis*. Semin Immunol. 2014;26(6):431-44. doi: 10.1016/j.smim.2014.09.012. PubMed PMID: 25453224; PubMed Central PMCID: PMC4314449.

12. de Jong BC, Hill PC, Brookes RH, Gagneux S, Jeffries DJ, Otu JK, et al. *Mycobacterium africanum* elicits an attenuated T cell response to early secreted antigenic target, 6 kDa, in patients with tuberculosis and their household contacts. J Infect Dis. 2006;193(9):1279-86. doi: 10.1086/502977. PubMed PMID: 16586366.

13. Bold TD, Davis DC, Penberthy KK, Cox LM, Ernst JD, de Jong BC. Impaired fitness of *Mycobacterium africanum* despite secretion of ESAT-6. J Infect Dis. 2012;205(6):984-90. doi: 10.1093/infdis/jir883. PubMed PMID: 22301632; PubMed Central PMCID: PMC3282571.

14. McGuire AM, Weiner B, Park ST, Wapinski I, Raman S, Dolganov G, et al. Comparative analysis of Mycobacterium and related Actinomycetes yields insight into the evolution of *Mycobacterium tuberculosis* pathogenesis. BMC Genomics. 2012;13:120. doi: 10.1186/1471-2164-13-120. PubMed PMID: 22452820; PubMed Central PMCID: PMC3388012.

15. Gopinath K, Moosa A, Mizrahi V, Warner DF. Vitamin B(12) metabolism in *Mycobacterium tuberculosis*. Future Microbiol. 2013;8(11):1405-18. doi: 10.2217/fmb.13.113. PubMed PMID: 24199800.

16. Gueant-Rodriguez RM, Gueant JL, Debard R, Thirion S, Hong LX, Bronowicki JP, et al. Prevalence of methylenetetrahydrofolate reductase 677T and 1298C alleles and folate status: a comparative study in Mexican, West African, and European populations. Am J Clin Nutr. 2006;83(3):701-7. PubMed PMID: 16522920.

17. Oussalah A, Besseau C, Chery C, Jeannesson E, Gueant-Rodriguez RM, Anello G, et al. Helicobacter pylori serologic status has no influence on the association between fucosyltransferase 2 polymorphism (FUT2 461 G->A) and vitamin B-12 in Europe and West Africa. Am J Clin Nutr. 2012;95(2):514-21. doi: 10.3945/ajcn.111.016410. PubMed PMID: 22237057.

18. Tang X, Deng W, Xie J. Novel insights into Mycobacterium antigen Ag85 biology and implications in countermeasures for *M. tuberculosis*. Crit Rev Eukaryot Gene Expr. 2012;22(3):179-87. PubMed PMID: 23140159.

19. Nielsen M, Lund O. NN-align. An artificial neural network-based alignment algorithm for MHC class II peptide binding prediction. BMC Bioinformatics. 2009;10:296. doi: 10.1186/1471-2105-10-296. PubMed PMID: 19765293; PubMed Central PMCID: PMC2753847.
